# Cullin-3 regulates the renal baroreceptor machinery that controls renin gene expression

**DOI:** 10.1172/jci.insight.194075

**Published:** 2025-07-08

**Authors:** Daria Golosova, Gaurav Kumar, Ko-Ting Lu, Patricia C. Muskus Veitia, Ana Hantke Guixa, Kelsey K. Wackman, Eva M. Fekete, Daniel T. Brozoski, Justin L. Grobe, Maria Luisa S. Sequeira-Lopez, R. Ariel Gomez, Pablo Nakagawa, Curt D. Sigmund

**Affiliations:** 1 Department of Physiology and; 2 Cardiovascular Center, Medical College of Wisconsin, Milwaukee, Wisconsin, USA.; 3 Department of Pediatrics, Child Health Research Center, University of Virginia School of Medicine, Charlottesville, Virginia, USA.

**Keywords:** Nephrology, Vascular biology, Genetic diseases, Hypertension, Mouse models

## Abstract

Mutations in Cullin-3 (*CUL3*) cause hypertension (HTN). We examined the role of smooth muscle cell (SMC) CUL3 in the regulation of renin gene expression. Mice with SMC-specific *CUL3* deletion (S-CUL3-KO) developed severe HTN with paradoxically preserved levels of plasma angiotensin peptides and renal renin expression. Cre-recombinase was active in juxtaglomerular (JG) cells, resulting in decreased CUL3 expression. We evaluated components of the renin cell baroreceptor and revealed preserved Lamin A/C but decreased integrin β1 expression in S-CUL3-KO. We hypothesized that Rab proteins are involved in integrin β1 downregulation. Silencing either *Rab21* or *Rab5* in *CUL3*-deficient HEK293 cells increased integrin β1 protein. Coimmunoprecipitation revealed a direct interaction between Rab5 and CUL3. CUL3 deficiency increased Rab5, suggesting it is regulated by a CUL3-mediated mechanism and that CUL3 deficiency results in loss of Rab protein turnover, leading to enhanced integrin β1 internalization. We conclude that the loss of integrin β1 from JG cells impairs the mechanosensory function of the renin cell baroreceptor, which underlies the persistent renin expression observed in hypertensive S-CUL3-KO mice. These findings provide insights into the molecular mechanisms of HTN, revealing that dysregulation of Rab proteins and integrin β1 in the kidney due to *CUL3* deficiency contributes to the development of HTN.

## Abstract 2

Vascular-specific deletion of CUL3 causes severe HTN with paradoxically normal levels of renin expression as a result of impaired renin cell baroreceptor.

## Graphical abstract

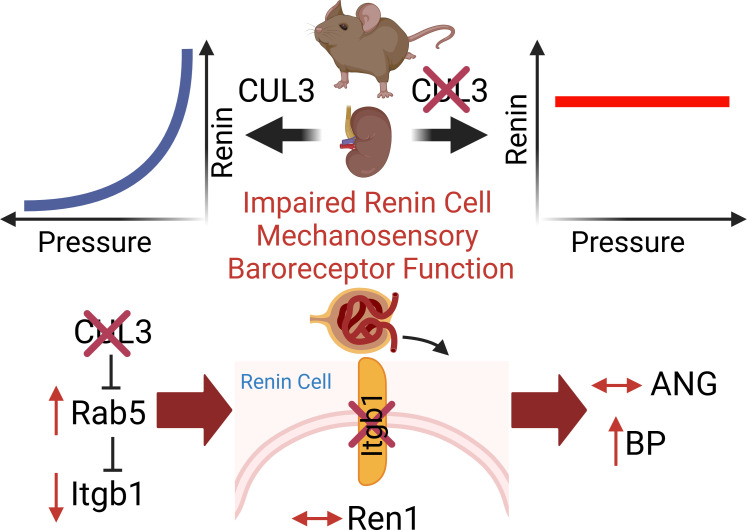

## Introduction

Mutations in Culin-3 (*CUL3*) were identified as a cause of hypertension (HTN) in humans (1). CUL3 is a component of the ubiquitin proteasome system, specifically within the family of Cullin-RING (Really Interesting New Gene) ubiquitin ligases (CRLs), which are involved in the regulation of protein turnover through ubiquitination (2). Among the CRLs, CUL3 plays a pivotal role in maintaining cellular homeostasis, and its dysfunction is implicated in several pathologies, including familial hyperkalemic HTN (FHHt), also known as pseudohypoaldosteronism type 2 or Gordon’s syndrome (3). FHHt is characterized by HTN, hyperkalemia, and metabolic acidosis. In the kidney, CUL3 mediates the activity of the thiazide-sensitive Na^+^-Cl^–^ cotransporter (NCC) in the distal convoluted tubule (DCT), a critical step in regulating sodium balance and blood pressure (BP) (4). Mutations in *CUL3*, its substrate adaptor Kelch-like protein 3 (*KLHL3*), and other associated kinases have been linked to aberrant phosphorylation and overactivation of NCC, contributing to the electrolyte abnormalities observed in FHHt (1, 5). Furthermore, CUL3 has been implicated in the degradation of the With No Lysine (WNK) kinases, which regulate NCC activity, highlighting its essential role in renal ion transport and BP control (3). All this emphasizes the central role of CUL3 in the regulation of electrolyte and acid-base balance.

Beyond the kidney, CUL3 is also involved in regulating vascular function. Previous studies have shown that mutations in the gene encoding Peroxisome Proliferator Activated Receptor Gamma (*PPARG*) lead to HTN in humans and mice (6, 7). Endothelial and vascular smooth muscle–specific expression of dominant negative mutations in *PPARG* cause increased susceptibility to HTN and vascular dysfunction (8–10). Mechanistically, this is partly due to reduced expression of CUL3 and Rho-related BTB domain containing 1 (RhoBTB1), a PPARγ target and CUL3 adaptor protein. Impaired expression of these proteins caused a dysregulation of Rho kinase signaling and disruption of nitric oxide signaling due to impaired degradation of phosphodiesterase 5 and Ras homolog gene family member A (RhoA), both of which are CUL3 target proteins (11–15). Indeed, conditional deletion of *CUL3* in vascular smooth muscle or expression of the human HTN-causing mutation in *CUL3* results in vascular dysfunction, increased arterial stiffness, and HTN (14, 15). We also showed that impairment of RhoBTB1 promotes arterial stiffness, which regresses rapidly upon restoration of RhoBTB1, specifically in vascular smooth muscle (12). Ultimately, the effects of CUL3 on BP appear to be mediated by an equal contribution from both kidney and vascular mechanisms (16, 17).

The renin-angiotensin system (RAS) is a highly conserved mechanism for controlling sodium homeostasis and BP. Renin serves as the rate-limiting step in the production of angiotensin II (ANG II). The synthesis and release of renin by the kidney is tightly controlled by a number of mechanisms, including the renal baroreceptor (18). The baroreceptor for regulating renin gene expression is located within the juxtaglomerular (JG) cells of the kidney. High perfusion pressure in the afferent arteriole suppresses renin, whereas low perfusion pressure stimulates renin expression and release from JG cells to maintain systemic BP (19). However, only recently have the molecular components of the renal baroreceptor been identified. Changes in perfusion pressure are sensed by integrin β1 and transmitted to the cell’s nucleus via Lamin A/C (20).

Mice with selective ablation of *CUL3* in vascular SMC exhibit robust HTN, which is driven by impaired regulation of the RAS. We hypothesize that this is due to impaired downregulation of renin gene expression, which should normally occur in response to severe BP elevation. Moreover, the impaired regulation of renin expression is mediated by dysfunction of the renal baroreceptor mechanism. Finally, we propose that CUL3 participates in the degradation of integrin β1, the key mechanosensor of the renal baroreceptor. This study represents the first investigation to our knowledge into the role of the renal baroreceptor in HTN in *CUL3*-deficient animals and the identification of components of renal baroreceptor, which may be regulated by the CUL3 pathway. Our study provides insights into the molecular mechanisms that link CUL3 to BP regulation and may partly explain the severity of HTN in patients with *CUL3* mutations.

## Results

**Figure 1:**
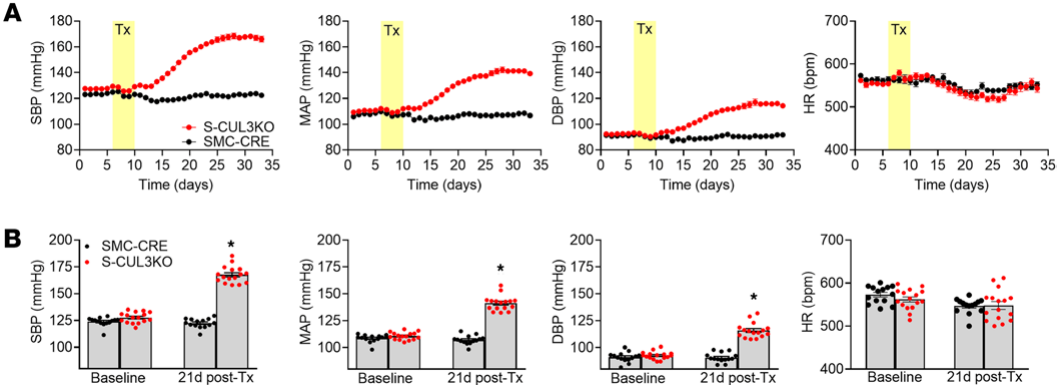
Blood pressure and heart rate in S-CUL3-KO mice. (**A**) Multiple cohort summary of systolic BP (SBP), mean arterial pressure (MAP), diastolic BP (DBP), and heart rate (HR). SMC-CRE, *n* = 14; S-CUL3-KO, *n* = 16. (**B**) Summary data for baseline BP and HR and maximal BP and HR after Tx in control and S-CUL3-KO mice. Data represent mean ± SEM. *P < 0.05 by 2-tailed independent *t* test.

We studied S-CUL3-KO mice generated by breeding mice expressing an inducible form of Cre-recombinase (CRE^ERT2^) driven by the myosin heavy chain promoter *Myh11* (termed SMC-CRE) with mice carrying a conditional *CUL3* allele (CUL3^fl/fl^) (15). S-CUL3-KO mice exhibited a progressive rise in BP over 21 days after administration of tamoxifen (Tx) compared with Tx-treated control SMC-CRE mice (Figure 1). Systolic blood pressure (SBP) peaked at 168 mmHg (compared with 124 mmHg in control) with similar increases in MAP (141 mmHg versus 109 mmHg) and DBP (116 mmHg versus 91 mmHg). This increase in BP represents some of the highest BP ever recorded in mice. The heart rate (HR) remained largely unchanged throughout the protocol and was not significantly different in SMC-CRE and S-CUL3-KO mice. We next performed a cosinor analysis to assess if there are alterations in the circadian rhythm of BP or HR in S-CUL3-KO mice (Supplemental Figure 1; supplemental material available online with this article; https://doi.org/10.1172/jci.insight.194075DS1). As expected, mesor was significantly higher for SBP in S-CUL3-KO mice after Tx. Tx-treated S-CUL3-KO mice exhibited a decrease in amplitude, suggesting a suppression of day-night changes, possibly indicating a loss of dipping responses. These mice also exhibited a decreased acrophase, indicating the peak SBP occurs earlier (~8 p.m.) compared with other groups (~1 a.m.). Similarly, S-CUL3-KO exhibit an altered bathyphase indicating the minimal SBP occurred (~8 a.m.) compared with (~1 p.m.) in the other groups. The only correlation between the cosinor analysis of SBP and HR was the reduction in amplitude in the Tx-treated S-CUL3-KO group.

Despite robust HTN, there was no apparent change in renal function as determined by the level of Na, Na/creatinine ratio, K, K/creatinine ratio, albumin, or albumin/creatinine ratio in urine (Supplemental Table 1), nor any change in transcutaneous glomerular filtration rate (tGFR) (Supplemental Table 2), suggesting a preservation of mechanisms autoregulating GFR. Moreover, neither tGFR nor albumin/creatinine ratio changed in S-CUL3-KO mice even after 27 weeks of Tx (data not shown). No differences in water and food intake were observed (Supplemental Table 3).

**Figure 2:**
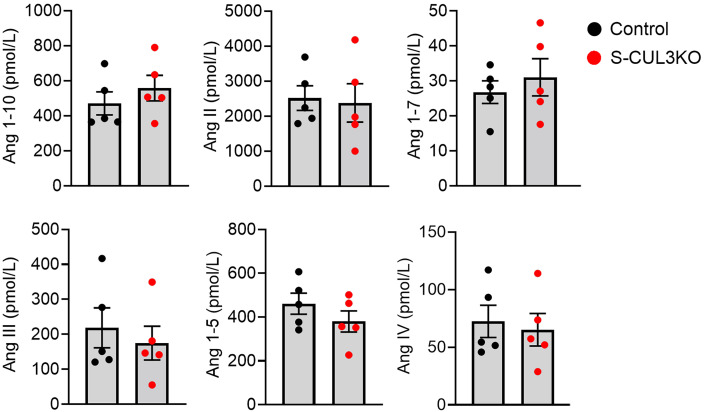
ANG peptides. Serum RAS fingerprint visualizing the equilibrium concentration of both the traditional and alternative RAS arms in hypertensive S-CUL3-KO (*n* = 5) compared with healthy controls (S-CUL3-KO + corn oil, *n* = 5). Data represent mean ± SEM. There were no significant differences detected by *t* test.

**Figure 3:**
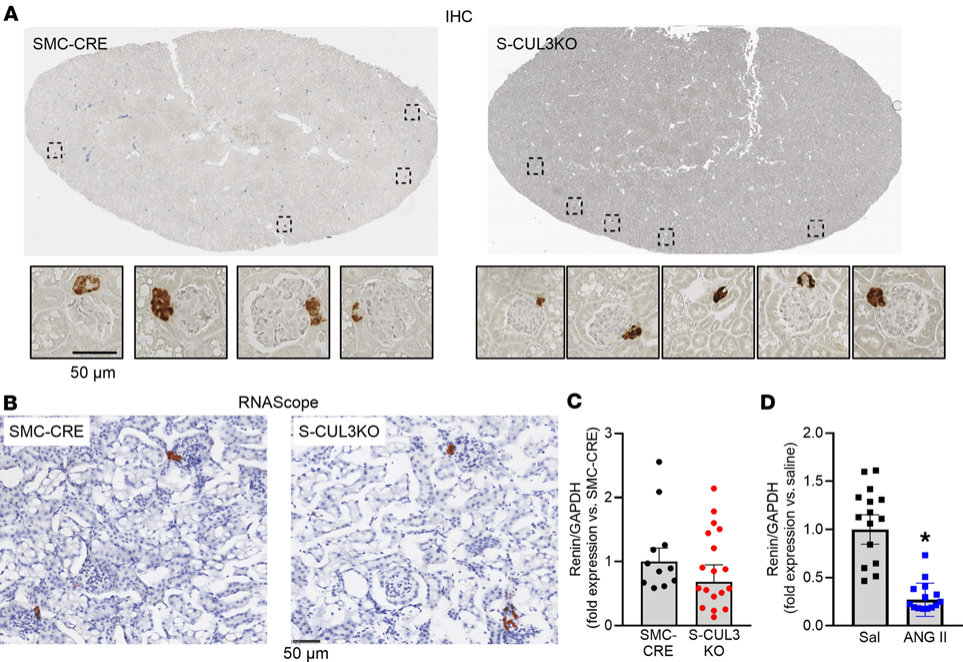
Renin expression in the kidney. (**A**) Representative IHC staining for renin protein in kidney slices in SMC-CRE and hypertensive S-CUL3-KO mice. Dotted boxes indicate the magnified pictures of JG apparatuses. Representative of 4–5 kidney sections per group. (**B**) In situ hybridization (*RNAscope*) for *Ren1* mRNA in the kidney. (**C**) *Ren1* mRNA as measured by qPCR of renal cortex mRNA 3 weeks after Tx administration. SMC-CRE, *n* = 11; S-CUL3-KO, *n* = 18. (**D**) *Ren1* mRNA as measured by qPCR of renal cortex of ANG II–treated (1,000 ng/kg of ANG II, 5 days) C57BL/6J mice. Data are shown as mean ± SEM, calculated by the ΔΔCT method. Saline, *n* = 15; ANG II, *n* = 14; *P < 0.05 by *t* test. Scale bars: 50 μm.

Previously, we showed that the levels of total ANG peptides in the plasma of S-CUL3-KO were unchanged (15). To examine this in more detail, we performed ANG peptide fingerprinting using mass spectroscopy. We measured RAS peptides after incubation of plasma at 37°C ex vivo, which provided the opportunity to measure each ANG peptide under physiologically relevant substrate concentrations in a manner similar to a plasma renin activity assay (21). Plasma was harvested 3 weeks after Tx administration at the peak of the HTN in S-CUL3-KO. Despite severe HTN, there were no differences in the levels of any of the ANG peptides assayed (Figure 2). With the preservation of ANG peptides, we next investigated if there was a change in the cell-specific expression of renin protein and mRNA. IHC revealed that renin protein expression was preserved within the JG apparatus in S-CUL3-KO mice, and that there was no qualitative difference in the level of JG renin (Figure 3A). In situ hybridization for *Ren1* mRNA similarly showed that renin mRNA was also localized exclusively to the JG area in S-CUL3-KO and that, qualitatively, there was no apparent reduction in the *Ren1* mRNA signal (Figure 3B). Although there was much greater variation in the level of *Ren1* mRNA in the kidney of S-CUL3-KO, quantitative PCR (qPCR) confirmed that *Ren1* mRNA levels in S-CUL3-KO were not significantly changed (*P* = 0.32) compared with SMC-CRE mice (Figure 3C). We performed a concurrent control where C57BL/6J mice were treated with a pressor dose of ANG II (1,000 ng/kg/day) which increased SBP (measured by radiotelemetry) to 151.5 ± 2.6 mmHg after 5 days. As expected, ANG II caused a robust decrease in *Ren1* mRNA in the kidney (*P* < 0.001) (Figure 3D). These data suggest that there may be an impairment in the regulation of *Ren1* expression in the kidney of S-CUL3-KO mice.

**Figure 4:**
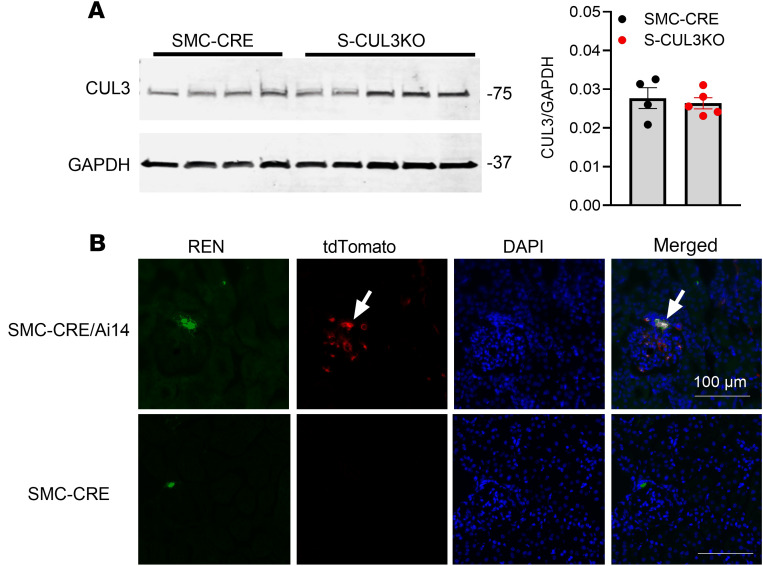
SMC-CRE activity in JG cells. (**A**) Representative Western blot showing expression of CUL3 protein in kidney cortex in SMC-CRE and S-CUL3-KO 3 weeks after Tx administration (left). Normalized CUL3 expression in SMC-CRE and S-CUL3-KO kidney (right). Data are presented as mean ± SEM. SMC-CRE, *n* = 4; S-CUL3-KO *n* = 5. *P < 0.05 by unpaired *t* test. (**B**) Cre activity was evaluated by tdTomato (red) expression with dual immunofluorescence targeting renin (green) in SMC-CRE X Ai14 reporter mice. Representative of 3–4 sections each from 2 separate kidneys per group.

**Figure 5:**
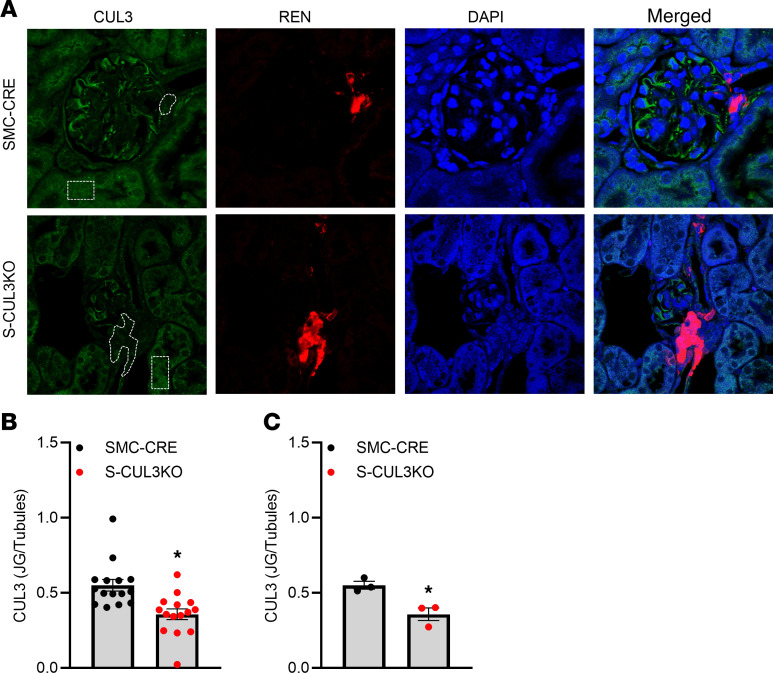
Decreased Cul3 expression in JG cells. (**A**) Immunofluorescence images detecting CUL3 (green) and renin (red) expression. Dashed lines indicate the regions used to quantify CUL3 expression. Round regions are renin-expressing regions, whereas squares represent non-renin-expressing neighboring tubules. Samples are derived from 3 independent biological replicates with 5 technical replicates per group. (**B** and **C**) Summary graphs demonstrating the level of CUL3 immunostaining in renin-expressing cells compared with neighboring non-renin-expressing tubules. **B** shows *n* = 3 biological replicates with *n* = 5 technical replicates/sample. **C** shows a summary of *n* = 3 samples averaged per section. Data represent mean ± SEM. *P < 0.05 by *t* test. Total original magnification, ×100.

Typically, an increase in BP results in a decrease in *Ren1* expression via the baroreceptor mechanism operating in JG cells (22). We hypothesized that an abnormality in *Ren1* expression may be caused by *Myh11*-driven Cre-recombinase activity in renin-expressing JG cells. First, CUL3 protein expression in kidney cortex was unaltered in S-CUL3-KO compared with SMC-CRE mice (Figure 4A). This was not surprising, given that CUL3 expression is ubiquitous, and SMC do not represent a very large population of total cells in the kidney cortex. Second, we crossed SMC-CRE with Ai14 [B6.Cg-Gt(ROSA)26Sor^tm14(CAG–tdTomato)Hze/J^] reporter mice to evaluate Cre activity in JG cells (Figure 4B and Supplemental Figure 2). Expression of tdTomato was employed as a surrogate for Cre activity and was clearly evident in vascular tissue and some glomeruli. Dual immunofluorescence staining revealed that tdTomato colocalized in renin-expressing cells, suggesting that JG cells are targeted in SMC-CRE mice. We next assessed if there was a reduction of CUL3 expression in renin-expressing cells, which was challenging because CUL3 expression is both ubiquitous and diffuse. We performed dual immunofluorescence for CUL3 and renin (Figure 5A and Supplemental Figure 3). Qualitatively, there appeared to be a decrease in the intensity of CUL3 immunofluorescence in renin-containing cells from S-CUL3-KO mice. To quantify this, the level of CUL3 immunostaining in renin-containing cells was compared with neighboring non-renin-expressing tubules. This analysis revealed a significant decrease in expression of CUL3 in renin-expressing cells when quantified either as level of expression per section (0.55 ± 0.04 in SMC-CRE versus 0.36 ± 0.04 in S-CUL3-KO, *P* = 0.001, *n* = 15 per group; Figure 5B) or averaged over the number of sections per mice (0.551 ± 0.026 in SMC-CRE versus 0.358 ± 0.042 in S-CUL3-KO, *P* = 0.012, *n* = 3 mice per group; Figure 5C). Taken together, these data provide evidence of Cre enzymatic activity within renin-expressing cells, which implies that, in a S-CUL3-KO model, CUL3 is targeted in JG cells. Consequently, we hypothesized that this may influence expression of CUL3 targets involved in the renal baroreceptor mechanism.

**Figure 6:**
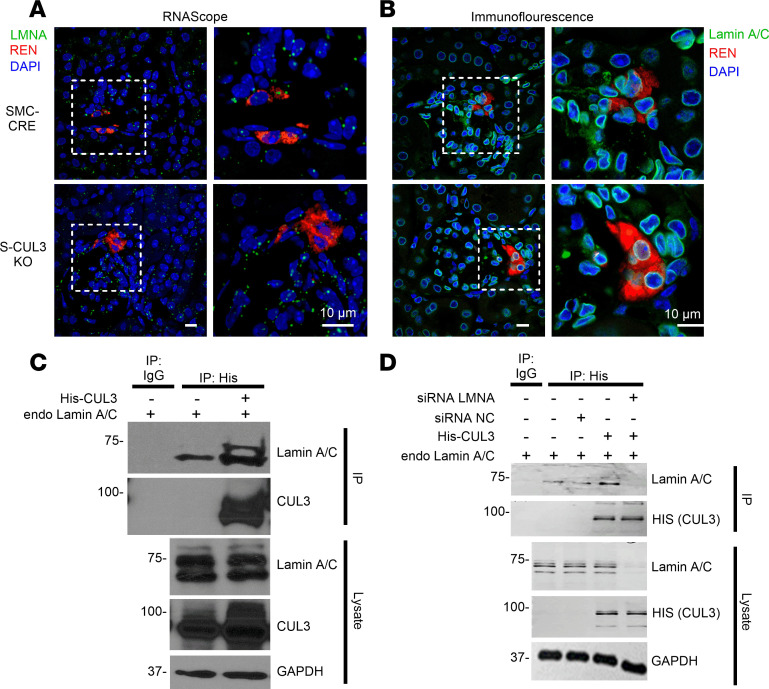
Renal Lamin A/C expression and interaction with CUL3. (**A**) Fluorescence in situ hybridization of kidney slices (RNAScope) for Lamin A/C (LMNA, green) and renin (red) counterstained with DAPI (blue). Dashed rectangles indicate JG area with expanded view to the right. SMC-CRE, *n* = 4; S-CUL3-KO, *n* = 3 biological replicates/group. (**B**) Immunofluorescence staining for Lamin A/C (green), renin (red), and DAPI (blue). Dashed rectangles indicate JG area with expanded view to the right. SMC-CRE, *n* = 4; S-CUL3-KO, *n* = 3 biological replicates/group. (**C**) Co-IP assay in HEK293 cells was performed with IgG or anti-His antibody and Western blotted with the indicated antibodies. Representative of 2 experiments. (**D**) Co-IP assay in A10 cells. IP was performed with IgG or anti-His antibody and Western blotted with the indicated antibody. The presence of nonspecific control (NC) or specific siRNA targeting LMNA is indicated. Representative of 2 experiments. Scale bars: 10 μm.

*Ren1* expression is regulated by the renin cell baroreceptor that decreases renin in response to increased perfusion pressure and increases renin when perfusion pressure decreases (20, 23). Thus, we next evaluated 2 major components of the renal baroreceptor mechanism, Lamin A/C and integrin β1, which were previously shown to regulate *Ren1* (20). Lamin A/C is important for the structural and functional integrity of renin-expressing cells and the regulation of gene expression in response to changes in perfusion pressure (20). Using in situ hybridization, we confirmed the presence of *Lmna* mRNA encoding Lamin A/C in renin-expressing cells of the kidney (Figure 6A and Supplemental Figure 4). Similarly, protein immunostaining revealed coexpression of renin and Lamin A/C protein in JG cells (Figure 6B and Supplemental Figure 5). Qualitatively, there did not appear to be any change in the level of Lamin A/C protein in renin-expressing cells from S-CUL3-KO compared with SMC-CRE. Nevertheless, we examined if a direct interaction between Lamin A/C and CUL3 exists by coimmunoprecipitation (co-IP) assays in HEK293 cells transfected with His-tagged CUL3. Specific Western blot bands corresponding to Lamin A/C and His-Cul3 after immunoprecipitation using anti-His coated beads (but nonspecific IgG) strongly suggest a direct interaction between His-CUL3 and endogenous Lamin A/C in HEK293 cells (Figure 6C). This was recapitulated in immortalized aortic rat smooth muscle A10 cells (Figure 6D). Importantly, Lamin A/C depletion but not the nonspecific scrambled control siRNA prevented immunoprecipitation of the complex. The absence of a change in the immunofluorescence signal for Lamin A/C in S-CUL3-KO was consistent with studies showing that the level of Lamin A/C protein did not change in response to MLN4924, a pan-Cullin inhibitor (data not shown). This suggests that Lamin A/C, although binding to CUL3, may not be targeted for degradation by the CRL3 pathway.

**Figure 7:**
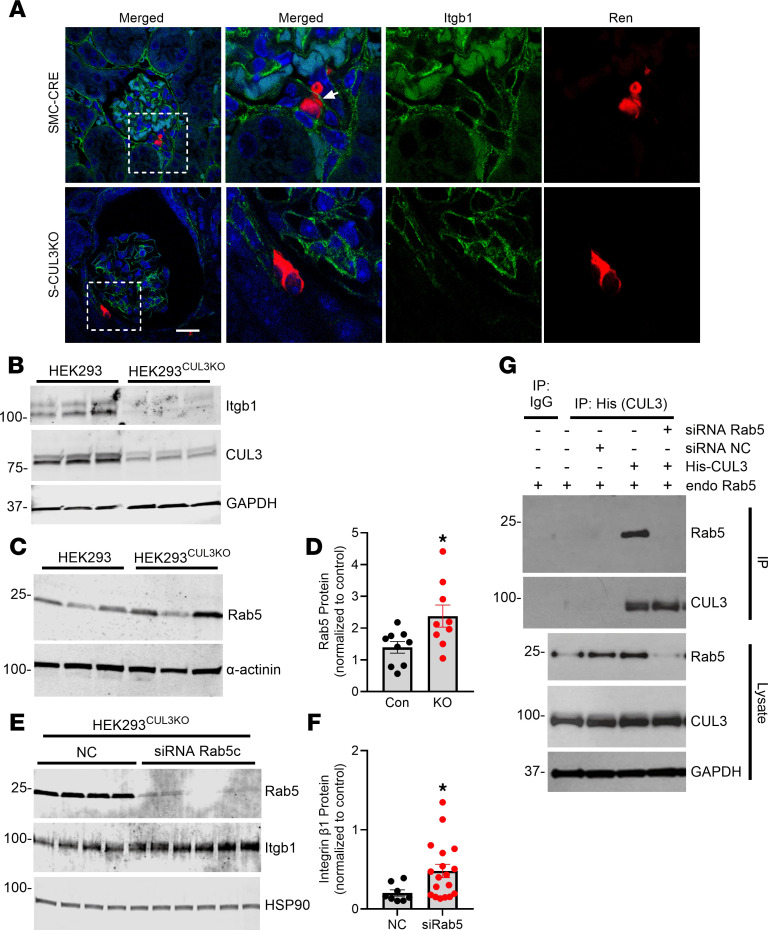
Renal integrin β1 expression. (**A**) Immunofluorescent probe demonstrating integrin β1 (*Itgb1*, green) and renin (*Ren*, red) protein expression in JG area and glomerulus in SMC-CRE and S-CUL3-KO cells. Dashed rectangle indicates the JG area. Magnified images are shown to the right. Scale bar: 10 μm. Arrow points to renin and integrin β1 coexpressing JG cell. Representative of 3–4 sections each from 3 (S-CUL3-KO) or 4 (SMC-CRE) kidneys from separate mice per group. (**B** and **C**) Western blotting in HEK293 and HEK293^CUL3KO^ cells reprobed as indicated. In **B**, the top blots were reprobed for GAPDH. In **C**, the Rab5 blot was reprobed for α-actinin. (**D**) Summary graph of quantitative changes in Rab5 expression; *n* = 9 (*n* = 6 compared with α-actinin, *n* = 3 with β-actin). Data represent mean ± SEM. *P < 0.05 by *t* test. (**E**) Western blotting HEK293^CUL3KO^ cells probed as indicated. (**F**) Summary graph of quantitative changes in Itgb1 expression. *n* = 8 for NC (nonspecific control siRNA); *n* = 18 for si*RAB5c*. Data represent mean ± SEM.*P < 0.05 by *t* test. (**G**) Co-IP assay in HEK293 cells. Cells were transfected with His-CUL3 for 16 hours and treated with siRNA targeting *Rab5c* or nonspecific control (NC) siRNA for 72 hours. IP was performed with IgG or antibody directed against the His epitope on the His-CUL3 fusion protein. Representative of 2 experiments.

In the JG apparatus, integrin β1 mediates the mechanical signaling from the afferent arteriole (20, 23). Thus, we evaluated integrin β1 protein in the JG region by immunofluorescence. In control SMC-Cre mice, integrin β1 protein was abundantly expressed in glomeruli and also was coexpressed with renin at the JG apparatus (Figure 7A and Supplemental Figure 6). S-CUL3-KO mice exhibited reduced integrin β1 in glomeruli and renin-expressing cells. We next measured the level of integrin β1 protein expression in HEK293 and HEK293^CUL3KO^ cells. HEK293^CUL3KO^ cells are *CUL3*-deficient HEK293 cells generated by CRISPR/Cas9 (24). As expected, HEK293^CUL3KO^ exhibited a marked decrease in CUL3 protein (Figure 7B). Consistent with the immunostaining, we also observed a significant reduction of integrin β1 protein expression in these cells.

Given the role of CUL3 as a ubiquitin ligase and protein turnover, the decrease in integrin β1 observed in CUL3 deficiency suggested an involvement of a CUL3-targeted intermediary protein regulating integrin β1. Because Rab proteins play a crucial role in regulating membrane trafficking of integrins, we hypothesized that CUL3 indirectly regulates the expression of integrin β1 via Rab protein turnover (25). We identified that Rab5, an early endosomal marker, was significantly increased in the HEK293^CUL3KO^ cells (Figure 7, C and D). We hypothesized that the enhanced recycling of integrin β1 from endosomes to the plasma membrane, regulated by endosomes, may account for a decrease in the level of integrin β1 in CUL3-deficient models. We next assessed if the decrease in integrin β1 was dependent upon Rab5, by targeting the c-isoform of *Rab5* mRNA with siRNA in HEK293^CUL3KO^ cells to evaluate the changes of integrin β1 protein. The siRNA was clearly effective, as there is a nearly complete reduction of total Rab5 protein (Figure 7, E and F). The decrease in Rab5 was accompanied by a significant increase in integrin β1 expression. These data indicate that the downregulation of Rab5 is an intermediate requirement for the impaired expression of integrin β1 due to CUL3 deficiency. Supporting this concept, endogenous Rab5 immunoprecipitates with CUL3 in His-CUL3 transfected HEK293 cells, suggesting a direct physical interaction between these 2 proteins (Figure 7G).

**Figure 8:**
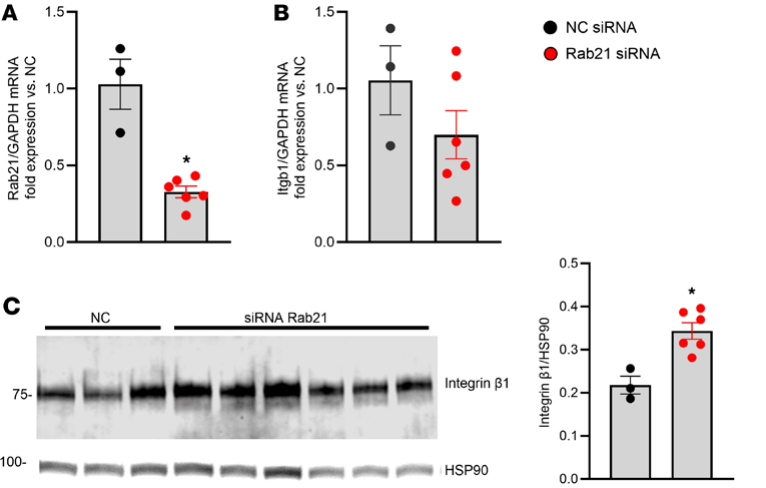
Role of Rab21 in integrin β1 in CUL3KO cells. (**A**) Expression of *Rab21* mRNA in response to *Rab21*-specific siRNA. *P < 0.05 by t test. (**B**) Expression of *Itgb1* mRNA in response to *Rab21*-specific siRNA. (**C**) Western blot for integrin β1 protein in HEK293^CUL3KO^ cells with control nonspecific or *Rab21*-specific siRNA. The graph summarizes the level of integrin β1. *n* = 3 for NC siRNA; *n* = 6 for *Rab21*-specific siRNA. Data represent mean ± SEM. *P < 0.05 by *t* test.

We also examined the related Rab7 protein. Like Rab5, Rab7 protein was increased in HEK293^CUL3KO^ cells. However, an siRNA targeting *Rab7* mRNA, which reduced *Rab7* mRNA by ~75%, was ineffective in changing the level of integrin β1 (data not shown). Since Rab21 plays a key role in regulating integrin trafficking, we also evaluated its effect on integrin β1 (26). *Rab21* mRNA was decreased ~70% after siRNA treatment in HEK293^CUL3KO^ cells (Figure 8A). Although *Rab21* silencing had no effect on expression of *Itgb1* mRNA (Figure 8B), it increased integrin β1 protein expression in HEK293^CUL3KO^ cells (Figure 8C). Because of a lack of efficient commercial antibodies against Rab21, we were not able to test if it was bound directly to CUL3 in vitro.

These data suggest that Rab proteins are CUL3-targeted master regulators of integrin β1 trafficking. Therefore, increased or prolonged expression Rab proteins implicates lower integrin β1 in renin-expressing JG cells, resulting in renal baroreceptor dysfunction. The preserved renin expression may result in the paradoxically normal level of ANG peptides, which may subsequently participate in the RAS-dependent component of HTN in S-CUL3-KO mice.

## Discussion

In this study, we investigated the effects of smooth muscle–specific deletion of *CUL3* on HTN, focusing specifically on paradoxically unaltered renal renin expression and circulating ANG peptides despite a marked increase in BP. We conclude that S-CUL3-KO mice exhibit an impaired pressure-sensing mechanism within renin-expressing JG cells of the kidney. Mechanistically, this appears to be mediated by a defect in the mechanotransducer machinery—notably, decreased levels of integrin β1 likely caused by increased internalization by Rab proteins. The lack of integrin β1 in renin-expressing JG cells fails to transduce transcriptional signals to appropriately regulate the renin gene, causing a failure to decrease renin expression despite very robust HTN. These data suggest that the machinery that regulates the renal baroreceptor and modulates renin gene expression may be under the control of CUL3-based mechanisms.

Renin is the rate-limiting enzyme within the RAS, where it functions to cleave angiotensinogen to produce ANG I. Renin synthesis and release by JG cells is tightly controlled by the levels of sodium chloride detected by the macula densa and the renovascular baroreceptor (27). S-CUL3-KO mice exhibit a progressive rise in BP, with SBP peaking at nearly 170 mmHg, 44 mmHg higher than SMC-CRE control mice. Although the reason BP peaks at 170 mmHg remains unclear, to our knowledge, this represents some of the highest BP reading reported in mice. For example, BP in BPH2 mice, which are commonly referred to as the mouse equivalent of the Spontaneous Hypertensive Rat (SHR), peak in the 145–150 mmHg range, roughly 20 mmHg higher than C57BL/6J mice (28). Development of HTN in S-CUL3-KO mice also exhibits a RAS-dependent and RAS-independent components, and peak BP is restored to normal after either ANG converting enzyme inhibition or ANG receptor blockade (29). The cosinor analysis of SBP seems to support the notion that the targeted animals are phase-shifted “left” in their circadian rhythm such that the highest pressures come at the beginning of the dark phase when activity and autonomic drive are expected to be the strongest. Moreover, the lack of a correlation between SBP and HR is consistent with the increased SBP in S-CUL3-KO mice occurring via increased total peripheral resistance, which is consistent with the design of the model in which CUL3 is specifically deleted in vascular smooth muscle.

Renin is subjected to a short feedback loop—namely, as BP increases, *Ren1* expression decreases. Therefore, given the significant BP increase observed in S-CUL3-KO, we hypothesized that we would observe reduced renin expression and a concomitant decrease in the level of downstream RAS effectors, ANG peptides. Contrary to our hypothesis, both *Ren1* mRNA and renin protein were both qualitatively and quantitatively preserved in the kidney, and the level of ANG peptides in plasma was preserved in S-CUL3-KO mice.

In adults, renin expression is restricted to a very small number of cells lining the afferent arteriole proximal to the glomerulus—thus their name, JG cells. During development, however, *Ren1* mRNA and renin protein can be detected in the growing and developing arterial tree (30). It has long been thought that the epithelioid-appearing, renin-expressing JG cells descend from a renin cell precursor that also gives rise to SMCs, mesangial cells, and pericytes (31, 32). Therefore, perhaps it was not surprising that Cre recombinase controlled by the *Myh11* promoter might be expressed in JG cells. Indeed, our data from a breeding of SMC-CRE with the Ai14 reporter revealed coexpression of tdTomato, the product of Ai14 recombination, with renin. Moreover, we observed a modest decrease of CUL3 expression in renin-expressing cells, suggesting that targets of the CUL3 pathway may exhibit impaired regulation. Our data suggest that components of the renin cell baroreceptor including Lamin A/C, a mechanotransducer that facilitates conformational changes of chromatin at the renin locus, and integrin β1, a mechanosensor that transduces changes of perfusion pressure signals to the JG cell, may be regulated by CUL3 (20). It remains unclear if the maintenance of *Ren1* expression in the kidney of S-CUL3-KO mice is due to a resetting of the baroreceptor to the higher BP established 21 days after CUL3 deletion. Arguing against this possibility is our data showing that C57BL/6J mice treated with a pressor dose of ANG II, which raises BP to a similar level as S-CUL3-KO mice, exhibited the expected downregulation of *Ren1* gene expression. Nevertheless, future studies aimed at either examining the components of the baroreceptor during the ramp to HTN in S-CUL3-KO mice or in response to increased pressure ramps in isolated kidneys could provide additional mechanistic insights (20, 33). In previously studied S-CUL3-KO mice, acetylcholine- and sodium nitroprusside–mediated vasorelaxation of the aorta was essentially normal 3 and 7 days after Tx. There was modest impairment at day 10 and significant impairment thereafter. This was consistent with the time course of the CUL3 deletion in the aorta of those mice in which significant loss of CUL3 protein was observed in aorta after 10 days of Tx. However, our attempt to generate a JG cell–specific KO of *CUL3* using Ren-CRE–knockin mice was thwarted by the fact that both the *CUL3* and *Ren1* genes are located on chromosome 1 in the mouse (32). We recognize that the absence of these studies represents a potential limitation of the current study. Future attempts to generate a renin-cell deletion of CUL3 may be performed using another Cre driver from the *Akr1b7* gene, which has also been reported to be specific for JG cells (34).

Interestingly, we did not observe any qualitative changes in the cell specificity or level of Lamin A/C expression in the kidney of S-CUL3-KO and SMC-CRE. However, we detected the interaction of Lamin A/C with CUL3 in our co-IP experiments using both HEK293 and A10 cells. The interaction was ablated by silencing of expression of the *Lmna* gene encoding Lamin A/C providing confidence in the specificity and selectivity of the IP assay. Moreover, we did not observe an increase in the level of Lamin A/C protein in cells treated with MLN4924, a pan Cullin inhibitor (data not shown). Therefore, if Lamin A/C is ubiquitinated by CUL3, it may not lead to its degradation. CUL3 has been reported to not only polyubiquitinate proteins leading to proteasomal degradation but also to monoubiquitinate proteins leading to their functional impairment (35, 36). Other mechanisms for Lamin A/C degradation have also been reported (37).

Since Lamin A/C is assembled and anchored to integrins, it became particularly intriguing to investigate integrins and explore how their dysfunction might impair this critical process. Integrins are a family of cell adhesion receptors that are used by cells to transduce structural and functional signals from the extracellular matrix into the cell (38). The ability of a cell to respond to mechanical forces is essential for multiple biological processes and signaling pathways. Integrins are endocytosed and recycled back to the plasma membrane, a process that plays a critical role in regulating cell adhesion, spreading, and motility (39, 40). Integrin αVβ5, for example, is internalized in an active, vitronectin-bound form through clathrin-coated pits and recycled back to the membrane pits.

Fibronectin is a ligand for integrin β1 and is highly expressed in renin cells (23). The application of fibronectin-coated magnetic beads to renin-expressing cells caused a decrease in *Ren1* mRNA expression, highlighting the effect of fibronectin-integrin interactions on *Ren1* gene regulation (20). It was also reported that integrin β1 functions as a mechanotransducer of the signaling changes produced by perfusion pressure in the model of aortic coarctation, suggesting its important role in mediating the renal baroreceptor–sensing mechanism.

Herein, we revealed a paradoxical decrease of integrin β1 levels surrounding renin-expressing cells in S-CUL3-KO kidneys. We also noted a qualitative redistribution of integrin β1 in the kidneys of S-CUL3-KO. Our observations revealed a distinct pattern of integrin β1 distribution, with concentrated localization predominantly at the cellular periphery coupled with significantly diminished expression throughout the cytoplasmic region. These alterations in integrin β1 expression were not limited to renin-expressing cells but also extended to additional glomerular structures, including podocytes. The decrease in integrin β1 expression was further validated in *CUL3*-deficient HEK293 cells. Integrin β1 has been reported to be regulated by CUL3 in endothelial cells (41). However, if integrin β1 were the direct target for CUL3-mediated degradation, we would have expected to see increased expression due to an impairment of proteasomal degradation. This prompted us to ask if there was another factor that could be responsible for the loss of integrin β1 in response to *CUL3* deficiency.

The Rab family of small GTPases regulates membrane trafficking, particularly of integrins through the binding to integrin cytoplasmic domains. The small GTPases Rab5 and Rab21 are particularly important in regulating integrin internalization and recycling (26). Ubiquitination of transmembrane protein cargos initiates endocytic trafficking, ultimately resulting in delivery to the lysosome (42). Components of the membrane-trafficking machinery, including Rabs, are also regulated by ubiquitination. Rab21 can be regulated by the ubiquitin-proteasome and autophagy-lysosome pathways (43). We demonstrated that silencing Rab5 expression facilitates the restoration of integrin β1 levels, suggesting that the loss of integrin β1 may be Rab5 dependent. Rab5 levels increased in *CUL3*-deficient HEK293 cells and coimmunoprecipitated with CUL3. Rab21 plays a role in integrin endocytosis and has been shown to directly interact with integrins (26). Knockdown of Rab21 impairs integrin-mediated cell adhesion and motility in human adenocarcinoma cells. Our studies also support the hypothesis that Rab21 might be involved in integrin β1 turnover, as silencing of Rab21 in *CUL3*-deficient HEK293 cells increased integrin β1 protein expression. These data suggest that accumulation of Rab5 or Rab21 in *CUL3* deficiency may have caused increase internalization of integrin β1, resulting in a loss of the necessary mechanotransducer on the surface of renin-expressing cells. Consequently, this dysfunction led to impaired integrin β1 expression, disrupting the mechanosensory mechanism of the renal baroreceptor, which preserved renin expression even in the concomitant presence of robust HTN. These data show that components of the renal baroreceptor regulating renin may be subjected to a complex posttranslational mechanism.

## Methods

### Sex as a biological variable.

Only male mice were studied because the *Myh11*-Cre^ERT2^ transgene is inserted on the Y chromosome (15).

### Experimental animals.

Mice carrying a conditional CUL3 allele (*CUL3^fl/fl^*, gift of Jeffrey Singer, Department of Biology, Portland State University, Portland, Oregon, USA) on a C57BL/6J genetic background were bred with mice expressing a smooth muscle–specific Tx-inducible Cre recombinase (*Myh11*-Cre^ERT2^) (15, 44). The Cre^+^/CUL3*^fl/fl^* mice (8–12 weeks of age) were treated with Tx (75 mg/kg, i.p. daily for 5 days) to promote smooth muscle–specific deletion of *CUL3* (S-CUL3-KO). Control mice were SMC-CRE mice treated with Tx. In 1 experiment, we used S-CUL3-KO mice treated with vehicle (corn oil) as a control group.

### BP measurements.

BP was continuously measured by radiotelemetry as we reported previously (14, 15). Mice were anesthetized with a mixture of ketamine (90 mg/kg i.p.) and xylazine (5 mg/kg i.p.) before a radiotelemetric BP transducer (PA-C10, Data Sciences International [DSI]) was implanted in the left common carotid artery. Analgesics were given during the surgery and again 24 hours later. After surgery, the mice were single housed and maintained on a heating pad overnight with free access to food (Teklad, 2920x) and filtered, chlorinated water. All animals were housed at room temperature (~22°C) under a 14:10-hour light-dark cycle (light onset at 5 a.m.). Animals were allowed to recover for at least 10 days before BP recordings. BP was continuously recorded for 10 seconds every 5 minutes for 24 hours. Data from each animal were averaged daily. BP was exported and analyzed using the Ponemah software (version 6.50; DSI). Water intake was monitored daily. Quality control metrics for the radiotelemetry included a minimum pulse pressure of 15 mmHg and preservation of the battery. Cosinor analysis was performed using a single 24-hour period (from noon to noon) each day at baseline and 21 days after Tx treatment using the Cosinor calculator (https://cosinor.online/app/cosinor.php) (45).

### ANG II infusion.

C57BL/6J littermates at 3–5 months of age received a 1-week infusion of either vehicle (0.15M NaCl in 0.01M acetic acid buffer) or ANG II (1,000 ng/kg/day, A9525, MilliporeSigma) via osmotic minipumps (model 2006, Alzet/Durect). Tissue harvest was performed at the end of the treatment.

### Quantification of the RAS.

RAS metabolite quantification was performed on snap-frozen plasma collected at the end of the experimental period. Analysis and quantification of steady-state levels of ANG peptides in equilibrated heparin plasma samples from the heart were performed by Attoquant Diagnostics according to the company’s protocol (21, 46). For equilibrium analysis, heparin plasma or serum is incubated at 37°C at a controlled pH level ex vivo, which allows the conversion of plasma angiotensinogen to ANG peptide through renin and other components of the RAS in the sample. Angiotensinogen concentration does not significantly change during incubation time, making renin the rate-limiting step of the reaction.

### Measurement of tGFR and urinalysis.

Briefly, tGFR was measured at the peak of HTN. An area of 2 × 2 cm of the dorsolateral skin was shaved, and then Nair was applied topically to remove hair. The fluorescent monitor was attached to the clean prepared area and secured with tape. After 3 minutes of baseline transmission under isoflurane anesthesia (2%), mice received FITC-sinistrin (0.07 mg per gram body weight; MediBeacon) via retroorbital injection. The dye injection took place immediately after the collection of baseline data; then, each mouse was immediately returned to its cage, where it could freely move under full consciousness. The clearance (removal rate) of FITC-sinistrin was monitored in the following 75 minutes using a transdermal fluorescent monitor (MediBeacon) for the calculation of tGFR. Additionally, urinary output was measured in metabolic cages for 24 hours. Urine electrolytes and creatinine were evaluated with a blood gas and electrolyte analyzer (ABL 800 Flex; Radiometer). Kidney function was also determined by measuring albuminuria using a fluorescent assay (Albumin Blue 580 dye, Molecular Probes) read by a fluorescent plate reader (FL600, Bio-Tek).

### Western blotting.

Protein lysates were extracted using RIPA buffer 50 mmol/L Tris Cl, 0.1 mmol/L EDTA (pH 7.5), 0.1 mmol/L EGTA, 1% w/v sodium deoxycholic acid, 1% w/v NP-40, and 0.1% w/v SDS, supplemented with protease (Roche, 11836170001) and phosphatase inhibitors (Roche, 4906845001). In total, 30 μg of protein lysates were separated using SDS-PAGE (4% to 20%) and transferred to PVDF membranes (MilliporeSigma, IPVH20200). After 1 hour of blocking in intercept blocking buffer (LI-COR Biosciences), membranes were incubated with primary antibodies at 4°C overnight followed by 1-hour incubation with horseradish peroxidase–conjugated secondary antibodies (LI-COR Biosciences, 926-32211 and 926-68070) at 1:20,000 dilution in blocking buffer. Blots were developed and imaged using LI-COR Western Sure ECL substrate and C-Digit Blot Scanner, respectively. LI-COR Image Studio Digits software (version5.2) was used for data acquisition. Primary antibodies used were: rabbit anti-Rab5 (Cell Signaling Technology, 3547, 1:1,000), rabbit anti-CUL3 (Cell Signaling Technology, A10450, 1:1,000), rabbit anti–integrin β1 (Cell Signaling Technology, 9699, 1:1,000), rabbit anti–α-actinin (Cell Signaling Technology, 3134, 1:1,000), mouse anti-GAPDH (Invitrogen, MA1-16757 1:1,000), rabbit anti-HSP90 (Cell Signaling Technology, 4877, 1:1,000), and anti-6X His-Tag antibody [HIS.H8] (Abcam, ab18184, 1:1,000).

### Cell culture.

HEK293 cells (CRL-1573, ATCC, USA) were cultured in DMEM (11885084, Thermo Fisher Scientific) supplemented with 5% FBS (10082147, Thermo Fisher Scientific) and 1% penicillin-streptomycin (15140122, Thermo Fisher Scientific) and maintained at 37°C with 5% CO_2_ as described previously (47).

A10 cells were purchased from the American Type Culture Collection (ATCC) cultured in DMEM (ATCC, 30-2002) supplemented with 5% FBS (Thermo Fisher Scientific, 10082147) and 1% penicillin-streptomycin (Thermo Fisher Scientific, 15140122) and maintained at 37°C with 5% CO_2_ as described previously (48). Dulbecco’s PBS (Thermo Fisher Scientific, 14190144) was used to wash cells prior to harvesting with 0.05% trypsin-EDTA (Thermo Fisher Scientific, 25300054).

### Co-IP.

Cells were transfected with siRNA 72 hours. Cell lysates were washed with ice-cold Dulbecco’s PBS and then mixed with IP lysis buffer containing 20 mmol/L Tris-HCl (pH 7.5), 150 mmol/L NaCl, 1 mmol/L Na_2_EDTA, 1 mmol/L EGTA, 1% Triton X-100, 2.5 mmol/L sodium pyrophosphate, 1 mmol/L β-glycerophosphate, 1 mmol/L Na_3_VO_4_, 1 μg/mL leupeptin, and 1 mmol/L PMSF. The cells were gently rotated in 1 mL centrifuge tubes for 10 minutes at 4°C, and the supernatants were collected by centrifuging at 13,000*g* for 10 minutes at 4°C. Next, 1 mg of the protein lysates was incubated with 20 μL of His-tag mouse antibody magnetic bead conjugate (Cell Signaling Technology, 8811S) overnight at 4°C with gentle rotation. The beads were separated using a magnetic rack and washed 3 times (5 minutes per wash) with IP lysis buffer at 4°C with gentle rotation. The immunocomplexes were then dissociated by boiling in 2× sample buffer. Immunoprecipitated proteins were resolved by SDS-PAGE and transferred to a nitrocellulose membrane (88018, Thermo Fisher Scientific). Membranes were blocked in 3% BSA in 0.1% Tris-buffered saline with Tween 20 (TBST) for 1 hour at room temperature, before being incubated overnight at 4°C with the appropriate primary antibodies. After washing the membranes 4 times with 0.1% TBST (5 min per wash), they were incubated with HRP-conjugated secondary antibody for 1 hour at room temperature. The membranes were washed again 4 times with 0.1% TBST, and protein bands were visualized using enhanced chemiluminescence reagents (1705060, Bio-Rad Laboratories) following the manufacturer’s instructions. Primary antibodies used were: rabbit anti-CUL3 (Cell Signaling Technology, A10450, 1:1,000), rabbit anti–integrin β1 (Proteintech, 12594-1-AP, 1:1,000), rabbit anti–Lamin A/C (Cell Signaling Technology, 2032S, 1:1,000), mouse anti-His (Invitrogen, Thermo Fisher Scientific, MA1-21315, 1:1,000), mouse anti–His-Tag (magnetic bead conjugate; Cell Signaling Technology, 8811S, 1:1,000), rabbit anti-His-Tag (Cell Signaling Technology, 2365S, 1:1,000).

### RNA interference.

A10 or HEK293 cells were transfected using Lipofectamine LTX-PLUS reagent (A12621, Thermo Fisher Scientific) according to the manufacturer’s protocol. All siRNA duplexes were transfected using Lipofectamine RNAiMax transfection reagent (13778075, Thermo Fisher Scientific) according to the manufacturer’s protocol. Silencing was performed by transiently transfecting cells with 100 nM DsiRNA for 72 hours. Predesigned DsiRNA were acquired from IDT (Integrated DNA Technologies) and OriGene Technologies. DsiRNA targeting *Rab5C* (hs.Ri.RAB5C.13.1, hs.Ri.RAB5C.13.2,hs.Ri.RAB5C.13.3), *Rab21* (SKU SR307933), and *Lmna* (rn.Ri.Lmna.13.1, rn.Ri.Lmna.13.2, rn.Ri.Lmna.13.3) with a concurrent negative control (NC) were supplied with a kit.

### Immunostaining.

Mice were euthanized with pentobarbital sodium (150 mg/kg, i.p.) and perfused transcardially with ice cold PBS supplemented with 2 mmol/L MgCl_2_ and 4% paraformaldehyde (PFA) in PBS (pH 7.4). Organs were dissected and postfixed in 4% PFA for 12 hours and then in 30% sucrose overnight. Kidneys were fixed, embedded, and processed as described previously (49). Tissue blocks were sectioned at 5 μm. Organs were randomized and coded before being submitted for blocking, sectioning, and staining. Researchers were blinded when performing the analysis. For chromogenic staining we used ImmPACT DAB Substrate Kit, Peroxidase (VectorLabs, SK-4105), and ImmPRESS HRP Polymer Detection Kit, Peroxidase (VectorLabs, MP-7405). Primary antibodies used were: rabbit anti–integrin β1 (Cell Signaling Technology, 34971, 1:100), goat anti-renin (Thermo Fisher Scientific, PA5-47607, 1:50), rabbit anti-CUL3 (Proteintech, 11107-1-AP, 1:50), and mouse anti–Lamin A/C (Cell Signaling Technology, 4777, 1:100). Secondary antibodies used were (1:400): donkey anti–goat Alexa-594 (Invitrogen, A-11058), goat anti–rabbit Alexa-594 (Invitrogen, A-32740), goat anti–rabbit Alexa Fluor 488 (Invitrogen, A-11008), and goat anti–mouse Alexa Fluor 488 (Invitrogen, A-11001).

### RNAscope in situ hybridization.

Kidneys S-CUL3-KO mice and control littermates were perfused with ice cold PBS followed by 4% PFA. After 24 hours fixation in 4% PFA, the organs and tissues were sectioned using a cryostat (Leica) at a thickness of 5 μm. RNAscope Multiplex Fluorescent V2 Assay (ACD Bio, 323110) was used to localize *Ren1* and *Lmna*. RNAscope 2.5 HD Assay – RED (ACD Bio, 322360) was used to localize *Ren1* in chromogenic kidney samples. Image acquisition and quantification were performed in a blinded fashion.

### qPCR.

Cell pellets were washed with 1× DPBS twice and snap frozen in liquid nitrogen. Kidney cortex and aorta were collected after heparin administration, and RNA was extracted using the TRIzol method (Invitrogen). RNA was isolated either by using PureLink RNA Mini Kit (Thermo Fisher Scientific, 12183018A) or TRIzol Reagent (Thermo Fisher Scientific, 15596026) according to the manufacturer’s protocol. RNA concentration was measured using a NanoDrop spectrophotometer with an OD260/OD280 ratio of greater than 1.9. Total RNA was reverse transcribed using SuperScript III reverse transcriptase (Invitrogen, 18080044), and qPCR was conducted using TaqMan Gene Expression Assays (Applied Biosystems). The assay numbers for TaqMan used in kidney are as follows: Mm02342887_mH (*Ren1*), Mm99999915_g1 (*Gapdh*). In HEK293 cells, they are: Hs01555790_g1 (*ITGB1*), Hs00209226_m1 (*RAB21*), and Hs02786624_g1 (*GAPDH*). The relative amount of mRNA was calculated after normalizing to its corresponding *GAPDH* using the 2^–ΔΔCT^ method (50).

### Statistics.

All data are presented as mean ± SEM. Parametric analyses were used throughout, including 2-way ANOVA with repeated measures and followed by selected (Šidák) or all pairwise (Tukey) multiple-comparison procedures and 2-tailed *t* test. *P* < 0.05 was considered statistically significant.

### Study approval.

All protocols were approved by the IACUC at the Medical College of Wisconsin. Surgical and experimental procedures adhered to the *Guide for the Care and Use of Laboratory Animals* (National Academies Press, 2011).

### Data availability.

Values for all data points in graphs are reported in the Supporting Data Values file. A file with raw blots is provided as supplemental material.

## Author contributions

DG designed research studies, conducted experiments, acquired and analyzed data, and wrote the manuscript. GK, KTL, PCMV, AHG, EMF, and DTB conducted experiments and acquired and analyzed data. KKW performed mouse husbandry. JLG analyzed data and wrote and edited the manuscript. MLSSL and RAG provided conceptual contributions and edited the manuscript. PN provided conceptual contributions, analyzed data, and edited the manuscript. CDS designed the research studies, acquired and analyzed data, provided reagents, and wrote and edited the manuscript.

## Supplementary Material

Supplemental data

Unedited blot and gel images

Supporting data values

## Version 1 07/08/2025

In-Press Preview

## Version 2 08/08/2025

Electronic publication
