# Supplementary material for: Cullin-3 regulates the renal baroreceptor machinery that controls renin gene expression
Source: JCI Insight. 2025 Jul 8;10(15):e194075. doi: 10.1172/jci.insight.194075 (PMC12333948; doi:10.1172/jci.insight.194075)
Supplement: Supplemental data [file jciinsight-10-194075-s099.pdf]

Supplemental Figures and Tables

Cullin-3 Regulates the Renal Baroreceptor Machinery that Controls Renin Gene Expression

Daria Golosova[1], Gaurav Kumar[1], Ko-Ting Lu[1], Patricia C. Muskus Veitia[1], Ana Hantke Guixa[1], Kelsey K. Wackman[1], Eva M. Fekete[1], Daniel T. Brozoski[1], Justin L. Grobe[1,2], Maria Luisa S. Sequeira-Lopez[3], R. Ariel Gomez[3], Pablo Nakagawa[1,2], Curt D. Sigmund[1,2].

[1] Department of Physiology, Medical College of Wisconsin, Milwaukee, WI

[2] Cardiovascular Center, Medical College of Wisconsin, Milwaukee, WI

[3] Department of Pediatrics, Child Health Research Center, University of Virginia School of Medicine, Charlottesville, Virginia

Corresponding Author:
Curt D. Sigmund
James J. Smith & Catherine Welsch Smith Chair of Physiology
Department of Physiology
Medical College of Wisconsin
8701 Watertown Plank Rd
Milwaukee, WI 53226
csigmund@mcw.edu

Supplemental Tables and Figures

Table S1: Kidney Function

Kidney Function	Control; n=10*	S-CUL3KO; n=15	P values
Diuresis, ml/day	0.67±0.13	0.49±0.05	0.2513
Na, mmol/day	0.15±0.02	0.15±0.03	0.9539
K, mmol/day	0.20±0.02	0.20±0.04	0.8759
Na/Creatinine	37±4	44±6	0.3408
K/Creatinine	52±7	57±4	0.5914
Albumin, mg/day	0.48±0.10	0.29±0.08	0.1469
Albumin/Creatinine	0.77±0.20	0.74±0.19	0.8938

*Control group – SMC-CRE+Tx or S-CUL3KO+corn oil

Table S1: Kidney function was measured six weeks after tamoxifen. P values are from a two-tailed T-test.

Table S2: Transcutaneous Glomerular Filtration Rate

Kidney Function	Control; n=11*	S-CUL3KO; n=11	P value
tGFR, µl/min	1067±76	1083±93	0.8893

Control group – SMC-CRE+Tx or S-CUL3KO+corn oil

Table S2: Transcutaneous glomerular filtration rate (tGFR) was measured three weeks after tamoxifen.

Table S3: Food and Water Intake.

Intake	Control; n=10*	S-CUL3KO; n=15	P value
Water, ml/day	3.69±0.33	4.03±0.39	0.4960
Food, mg/day	2.95±0.15	3.15±0.23	0.4807

*Control group – SMC-CRE+Tx or S-CUL3KO+corn oil

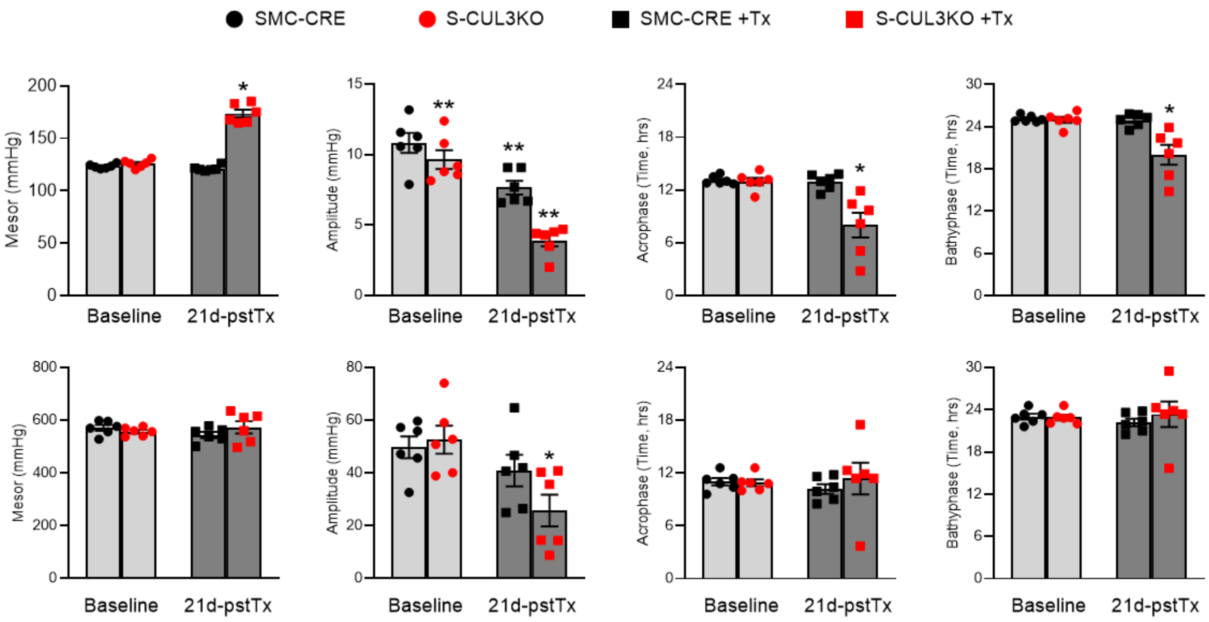

Figure S1. *Cosinor Analysis of BP and HR*. A cosinor analysis of SBP and HR was performed in a selection of 6 SMC-CRE and 6 S-CUL3KO at baseline and 21-days after Tx treatment. The same mice were analyzed before and after. *, P<0.05 S-CUL3KO +Tx vs SMC-CRE. **, P<0.05 all groups vs SMC-CRE.

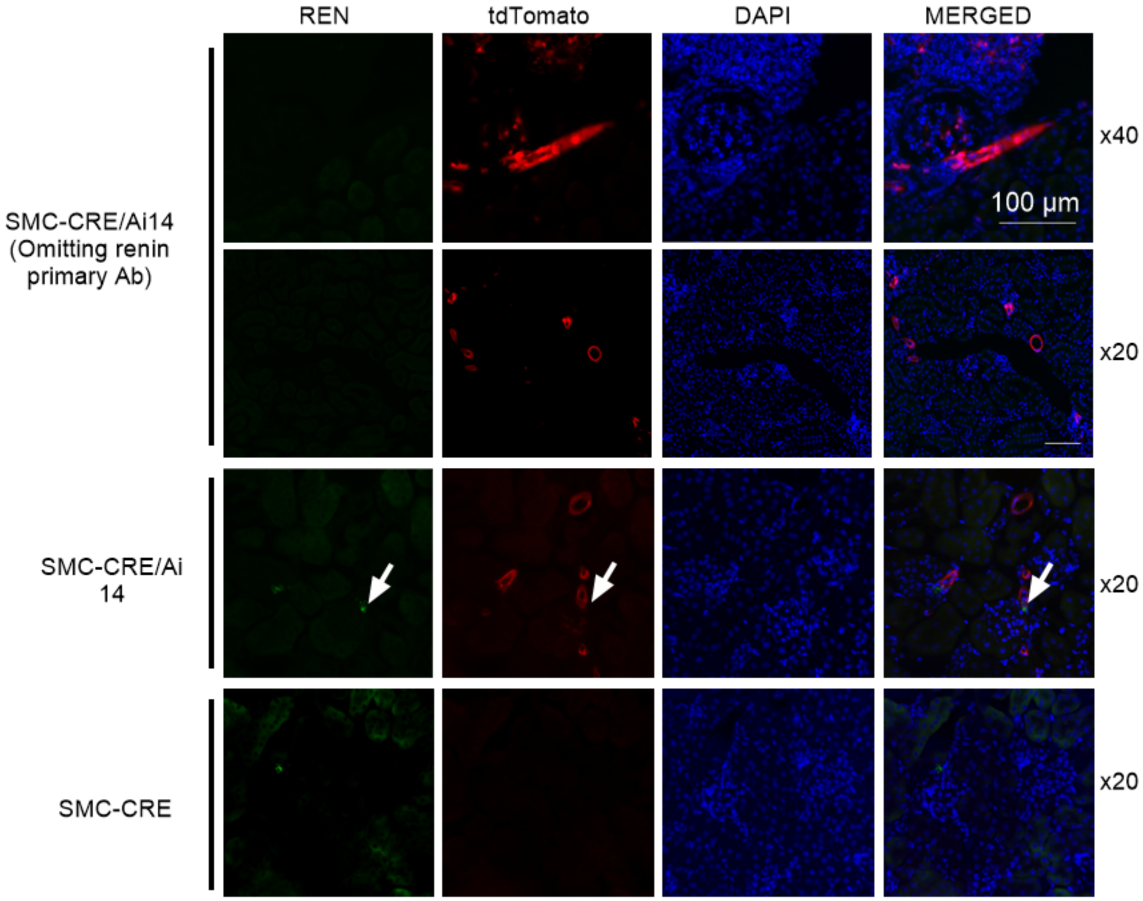

Figure S2. *SMC-CRE Activity in JG Cells*. Cre activity was evaluated by tdTomato (red) expression with dual immunofluorescence targeting *Ren1* (green) in SMC-CRE X Ai14 reporter mice. Top panel represents sections with the primary antibody targeting renin omitted. Bottom panels are additional sections illustrating Cre activity in renin-expressing JG cells.

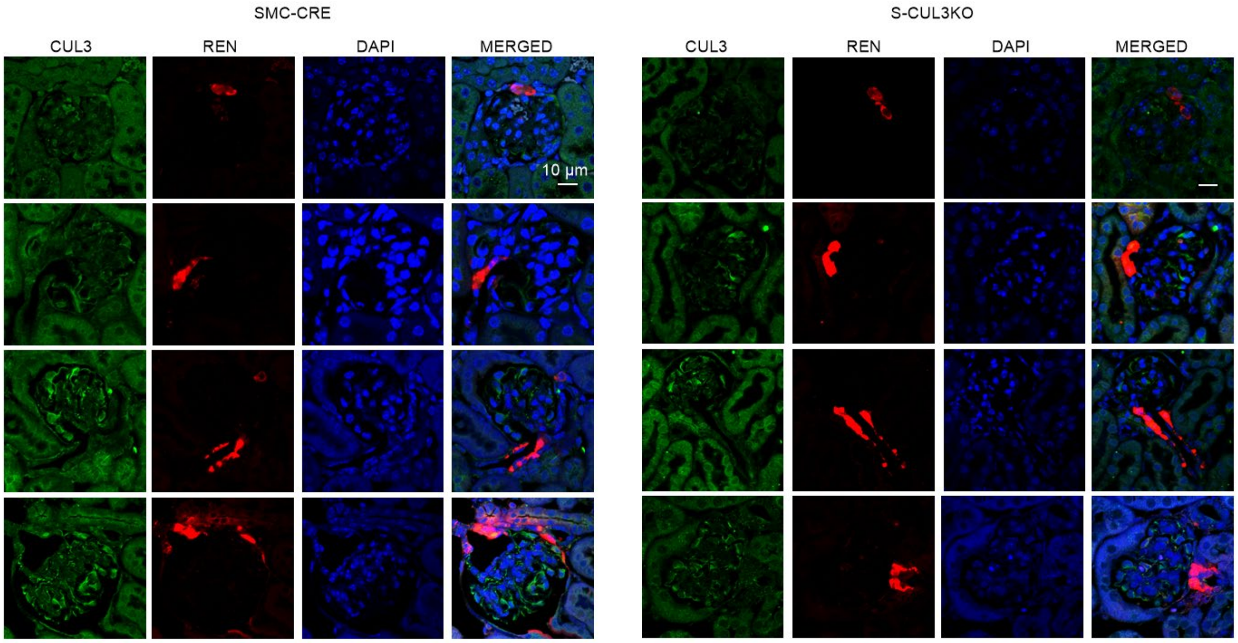

Figure S3. *Decreased Cul3 Expression in JG Cells*. Additional immunofluorescent images detecting CUL3 (green) and renin (red) expression.

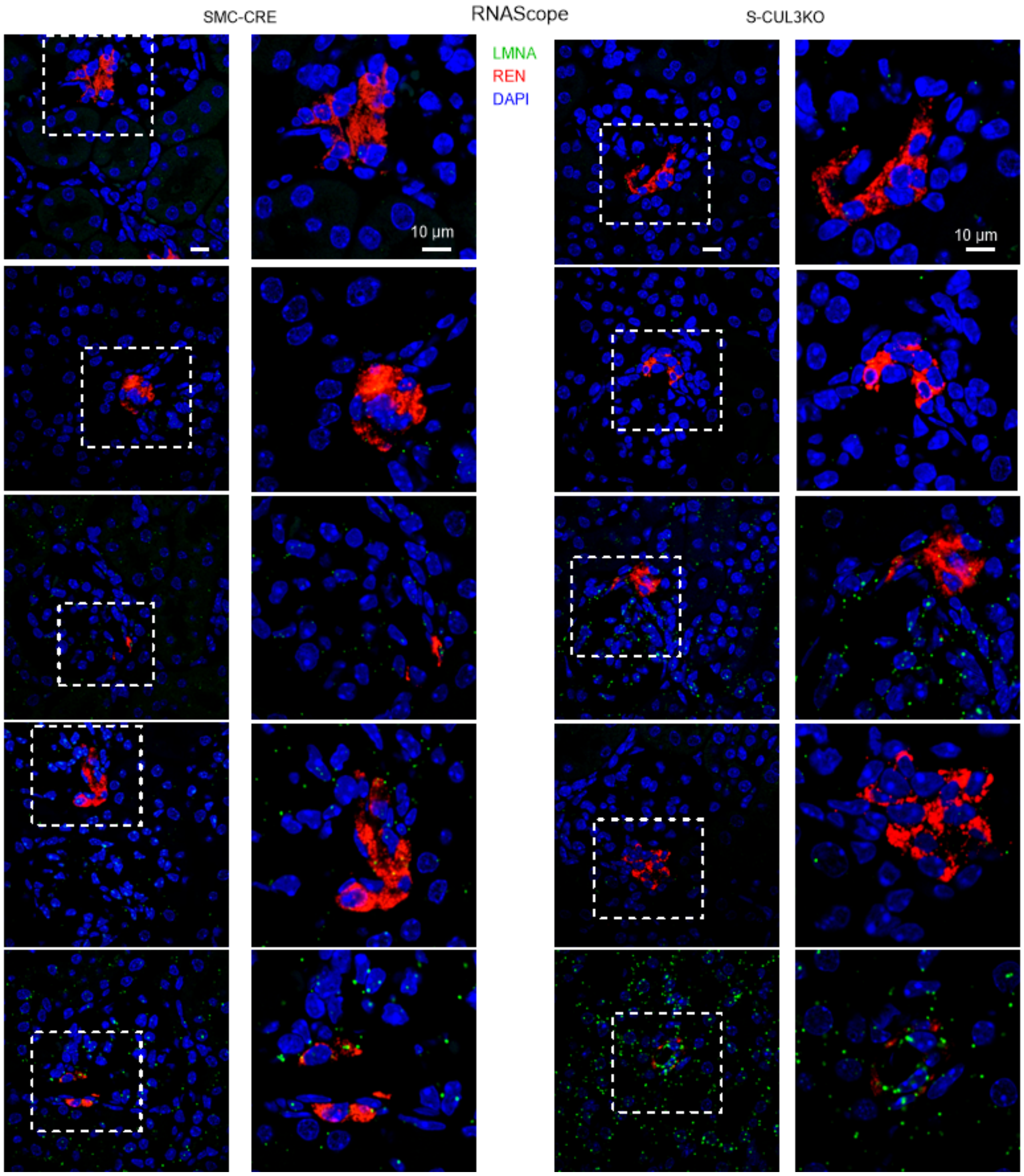

Figure S4. *Renal Lamin A/C Expression.* Additional representative RNAscope images demonstrating LMNA (green) and *Ren1* (red) mRNA in the JG area. Dashed rectangles indicate JG area with expanded view to the right.

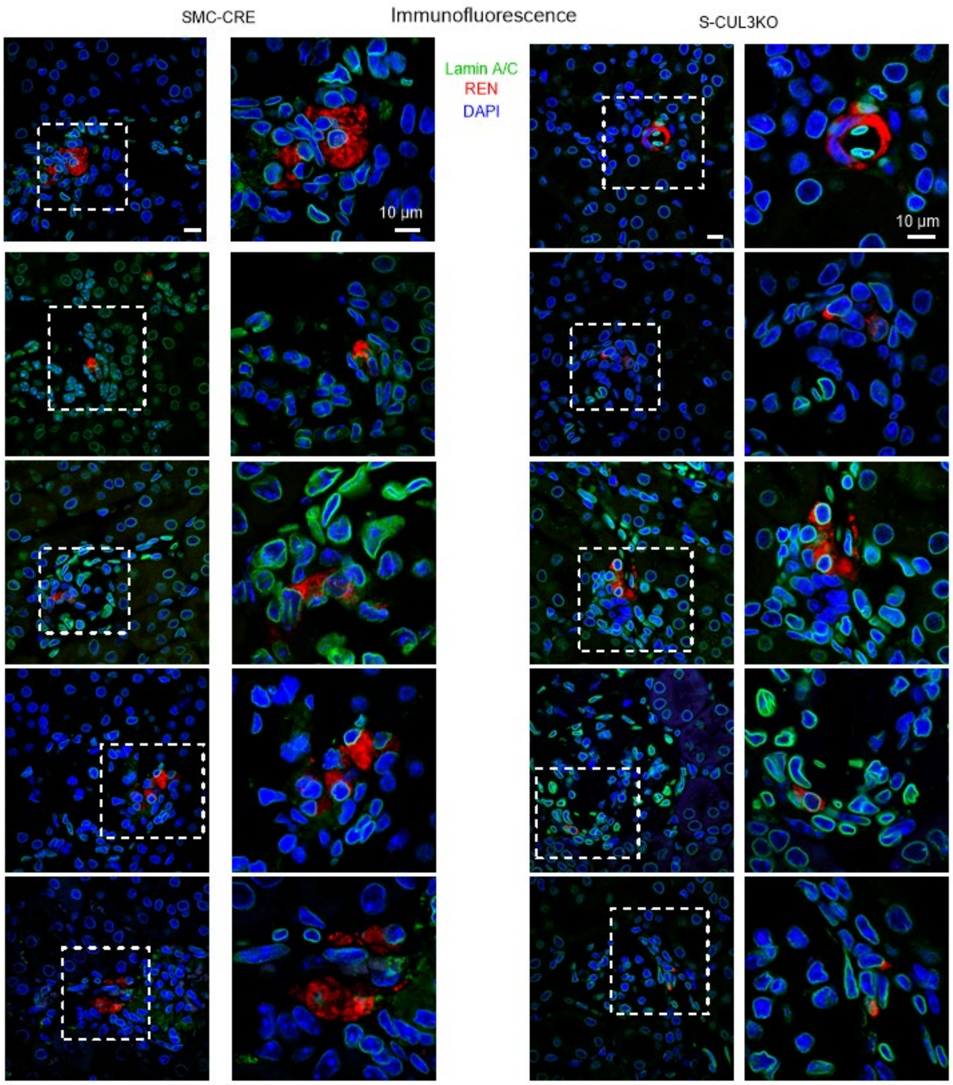

Figure S5. *Renal Lamin A/C Expression.* Additional representative immunofluorescent images demonstrating Lamin A/C (green) and *Ren1* (red) protein in the JG area

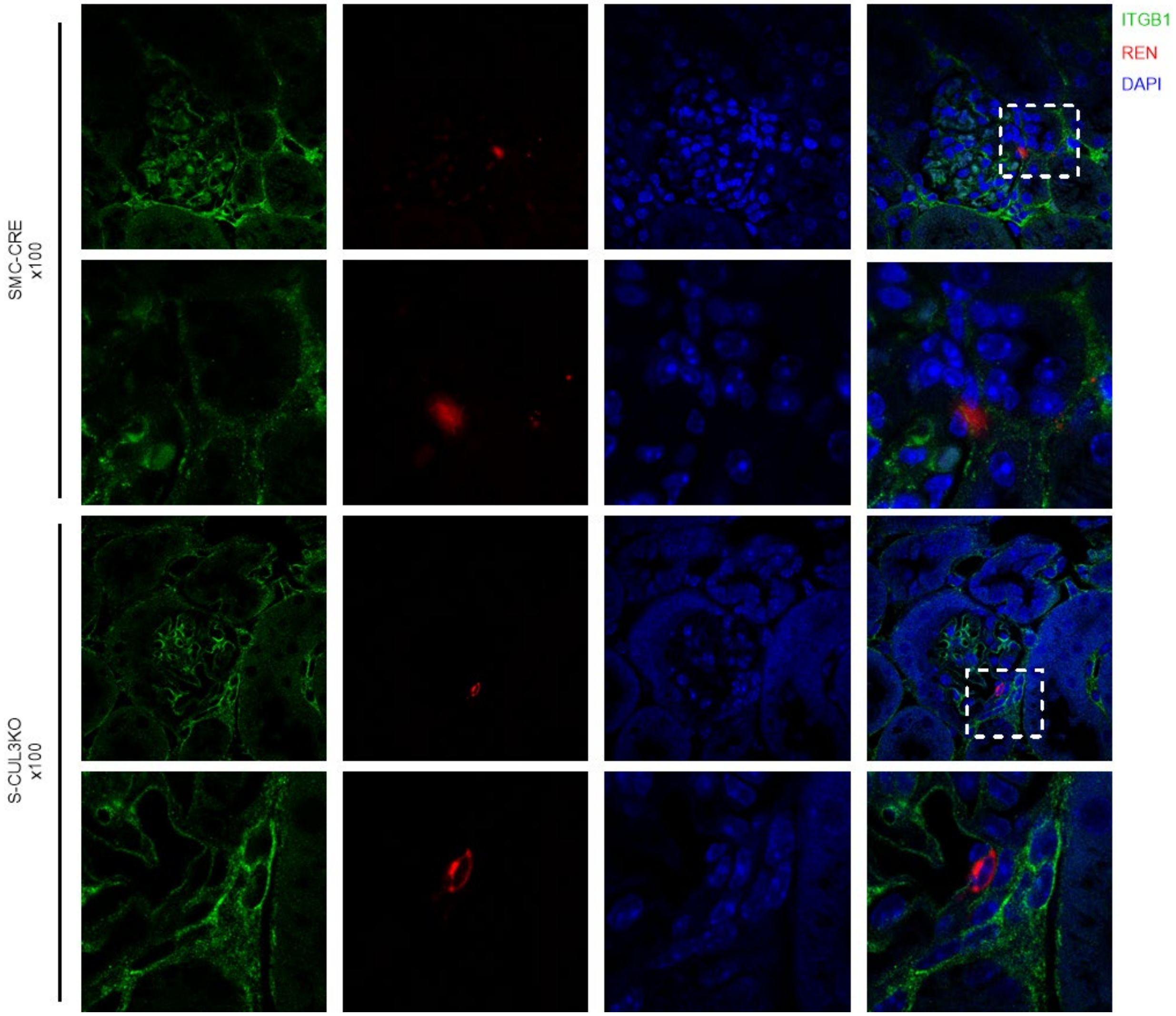

Figure S6. *Renal Integrin β1 Expression.* Additional immunofluorescent images demonstrating integrin β1 (green) and *Ren1* (red) protein in the JG area protein expression in the renin-expressing cells in the JG area, merged pictures. N=3-4/group
