# Supplementary material for: Cullin-3 regulates the renal baroreceptor machinery that controls renin gene expression
Source: JCI Insight. 2025 Jul 8;10(15):e194075. doi: 10.1172/jci.insight.194075 (PMC12333948; doi:10.1172/jci.insight.194075)
Supplement: Unedited blot and gel images [file jciinsight-10-194075-s100.pdf]

Full Unedited Blots

Figure 4

Full unedited blots for Figure 4A

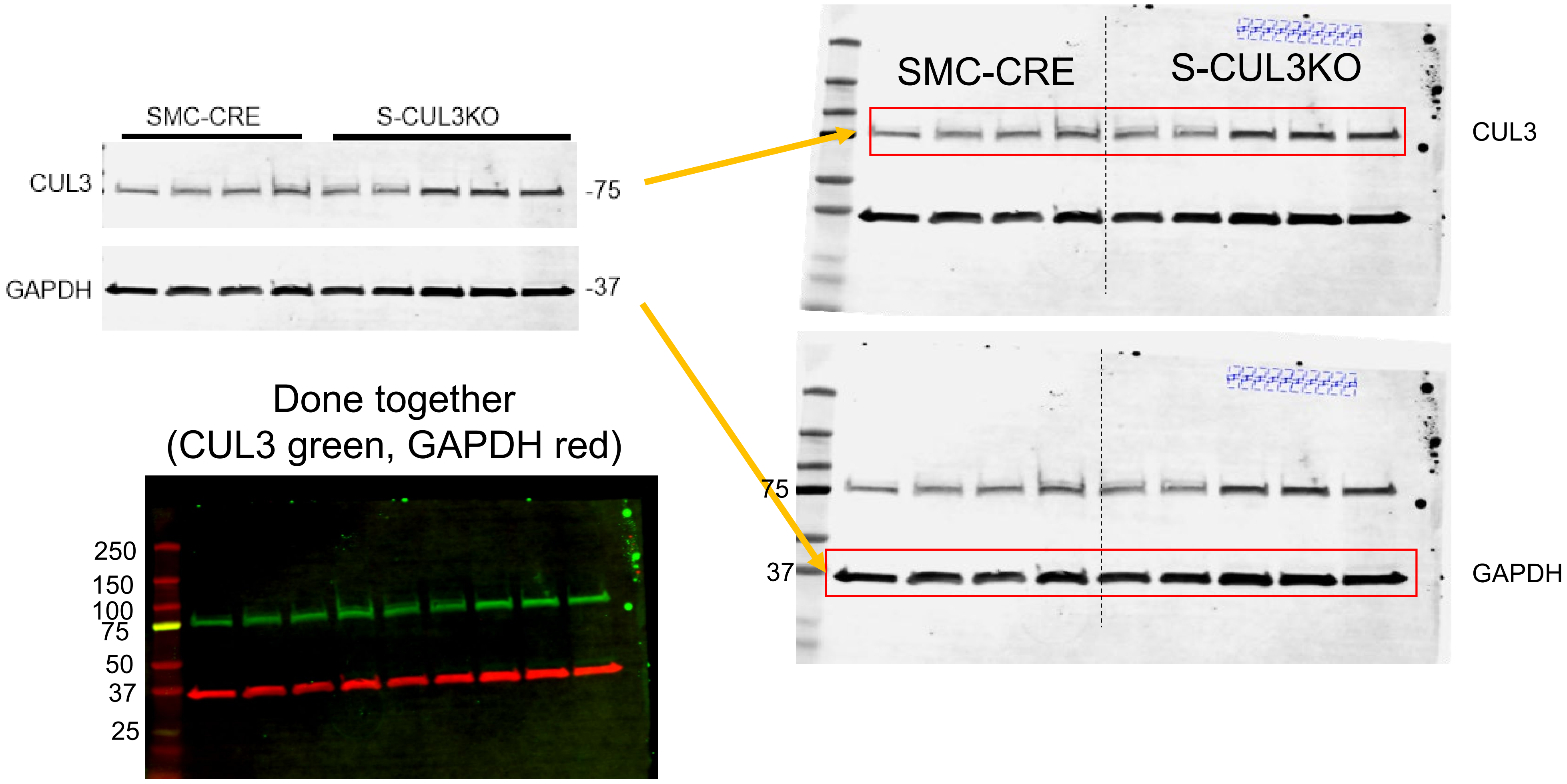

Figure 6C

Full unedited blots for Figure 6C

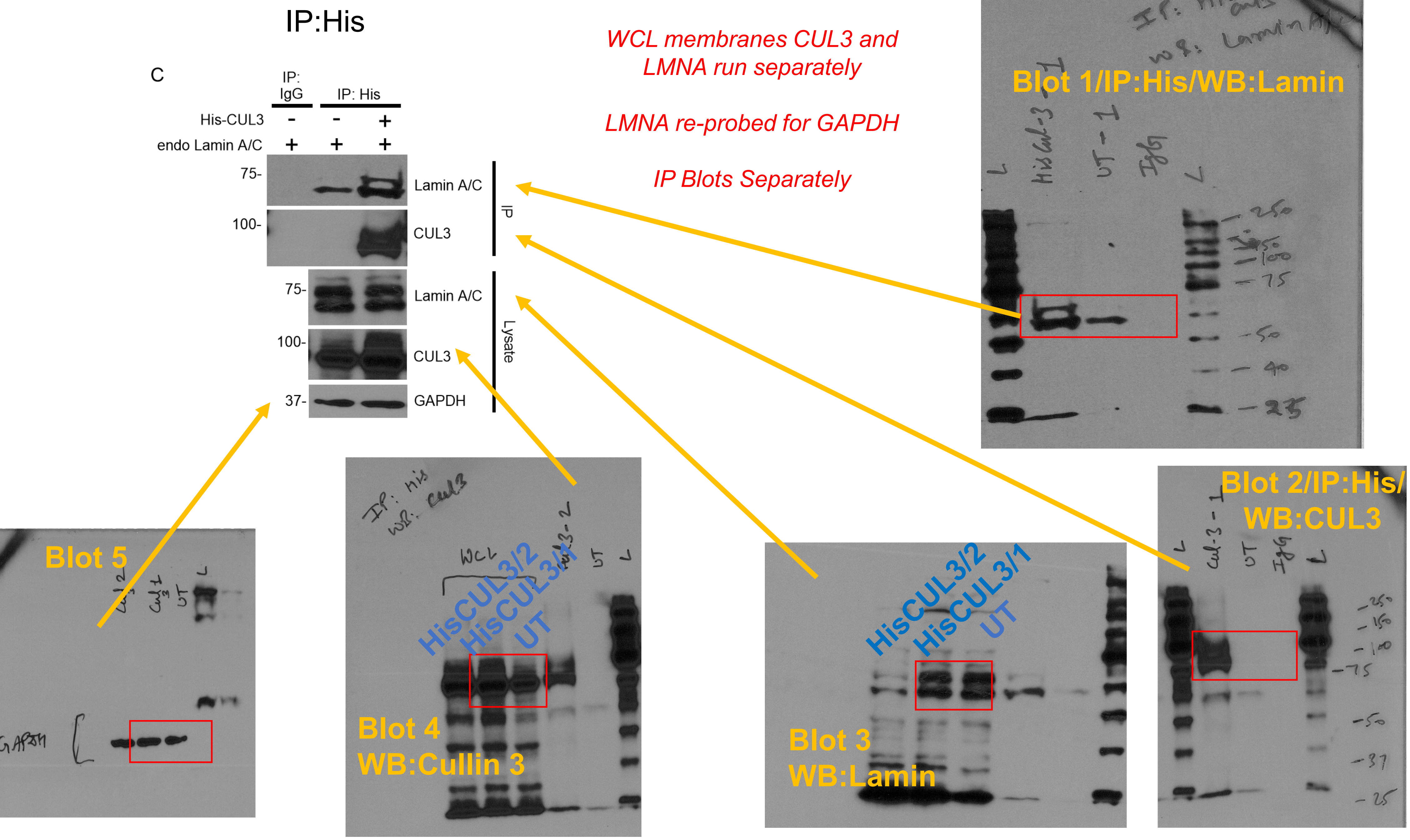

Figure 6D

Full unedited blots for Figure 6D

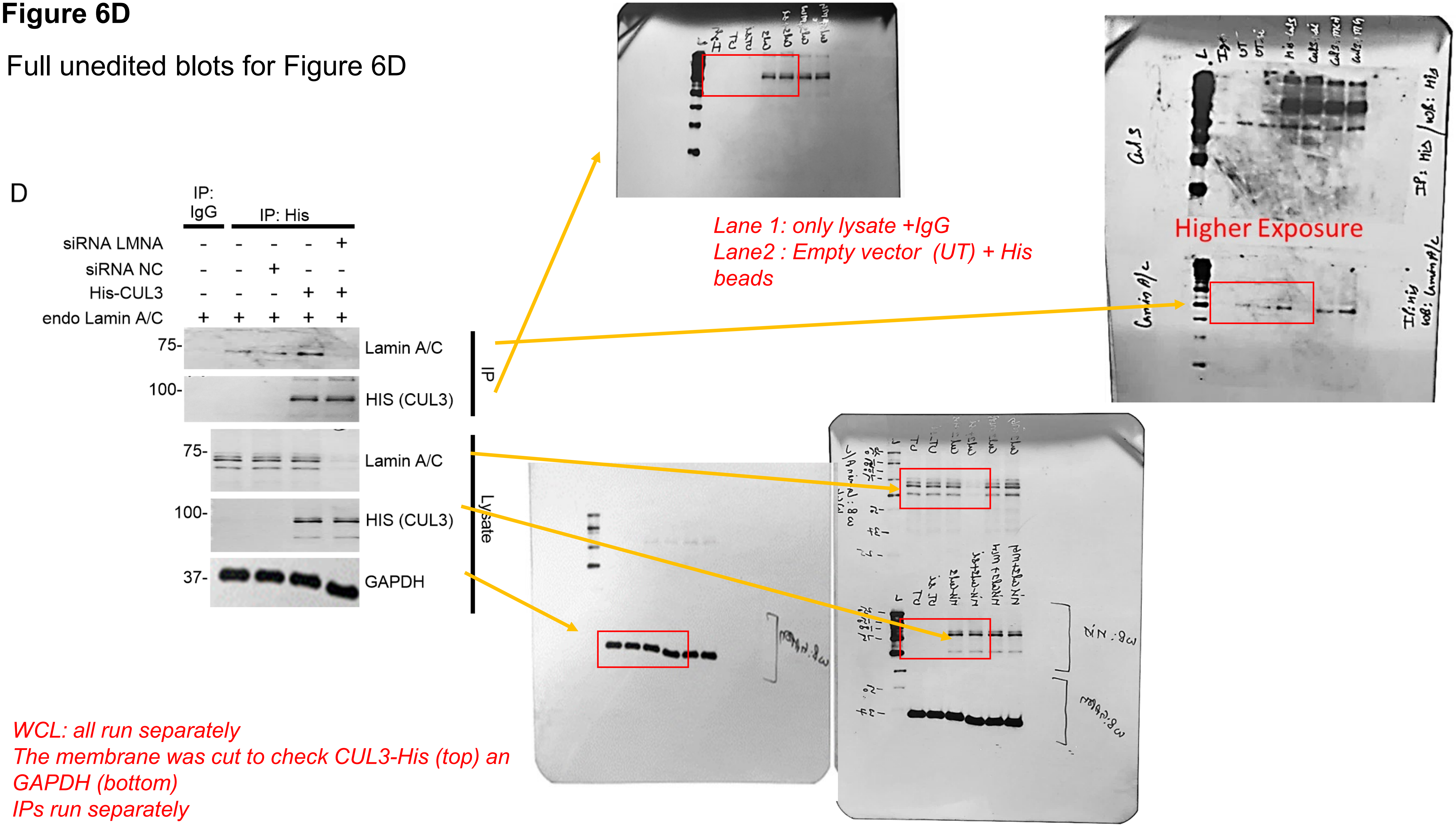

Figure 7B

Full unedited blots for Figure 7B

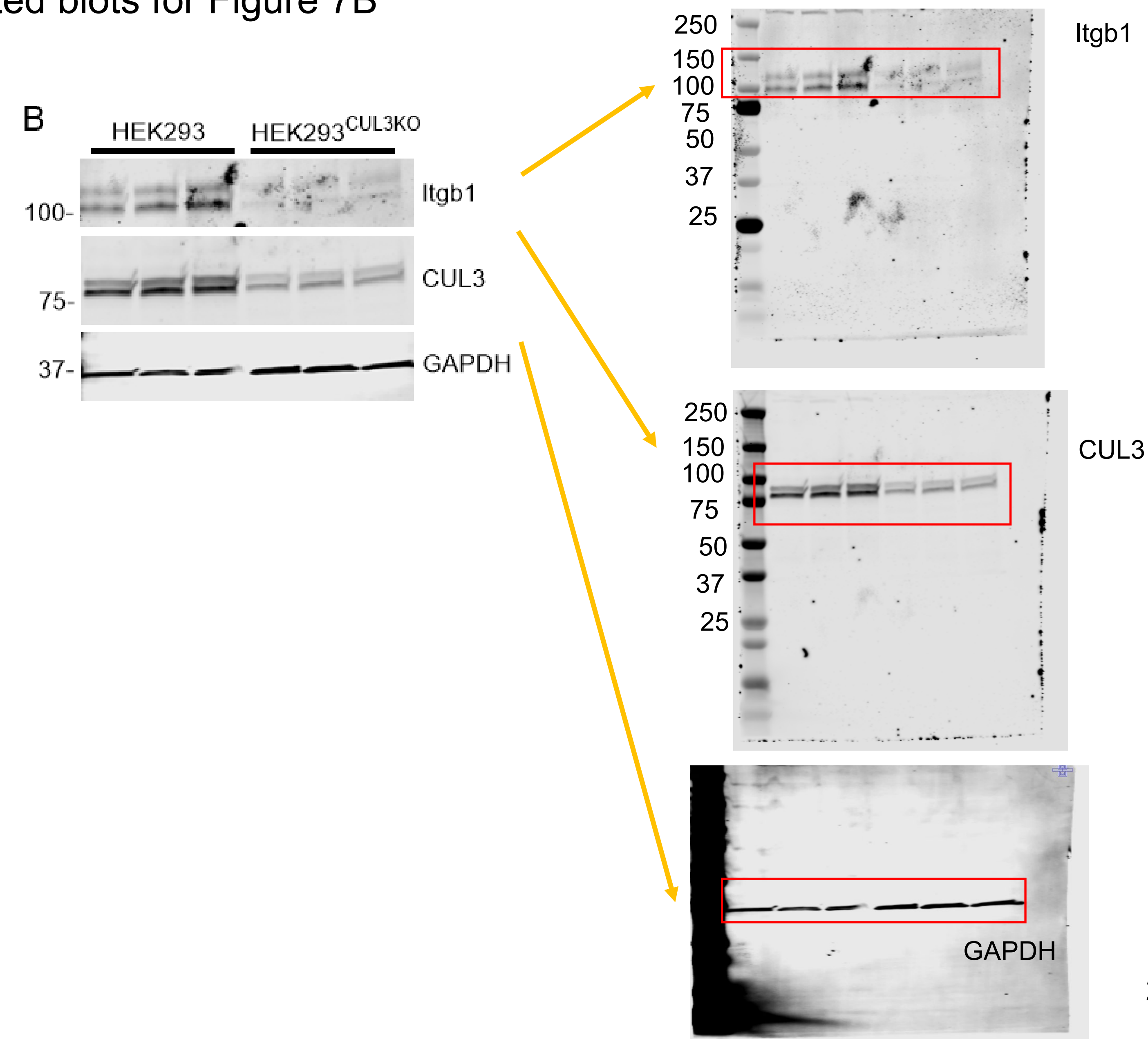

1) ITGB1+GAPDH together (LiCor 2 channels)
2) re-probed for CUL3

Figure 7C

Full unedited blots for Figure 7C

1) Rab5
2) a-Actinin

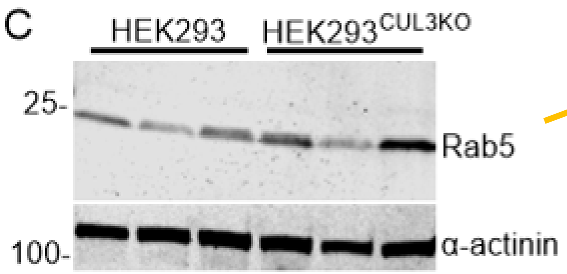

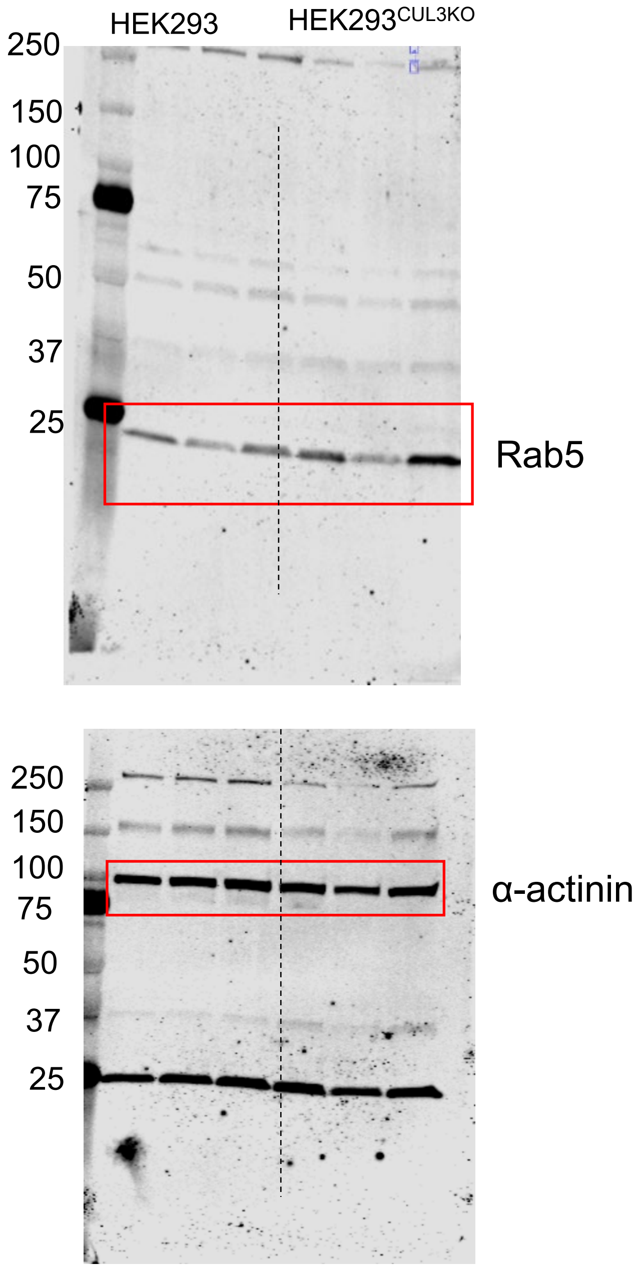

Figure 7E

Full unedited blots for Figure 7E

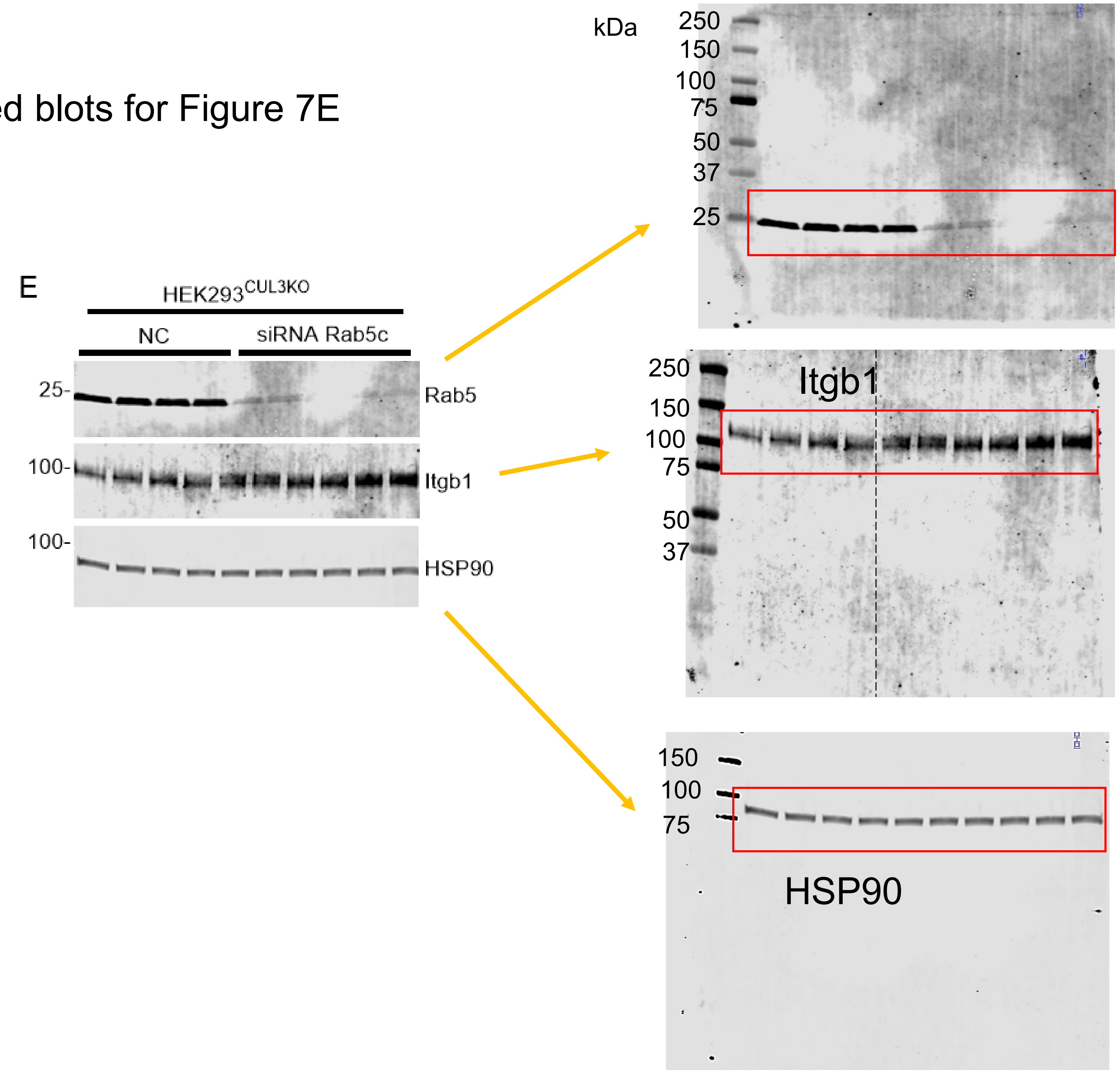

Order of probes.
1) Rab5
2) Itgb1
3) HSP90

Figure 7G Full unedited blots for Figure 7G

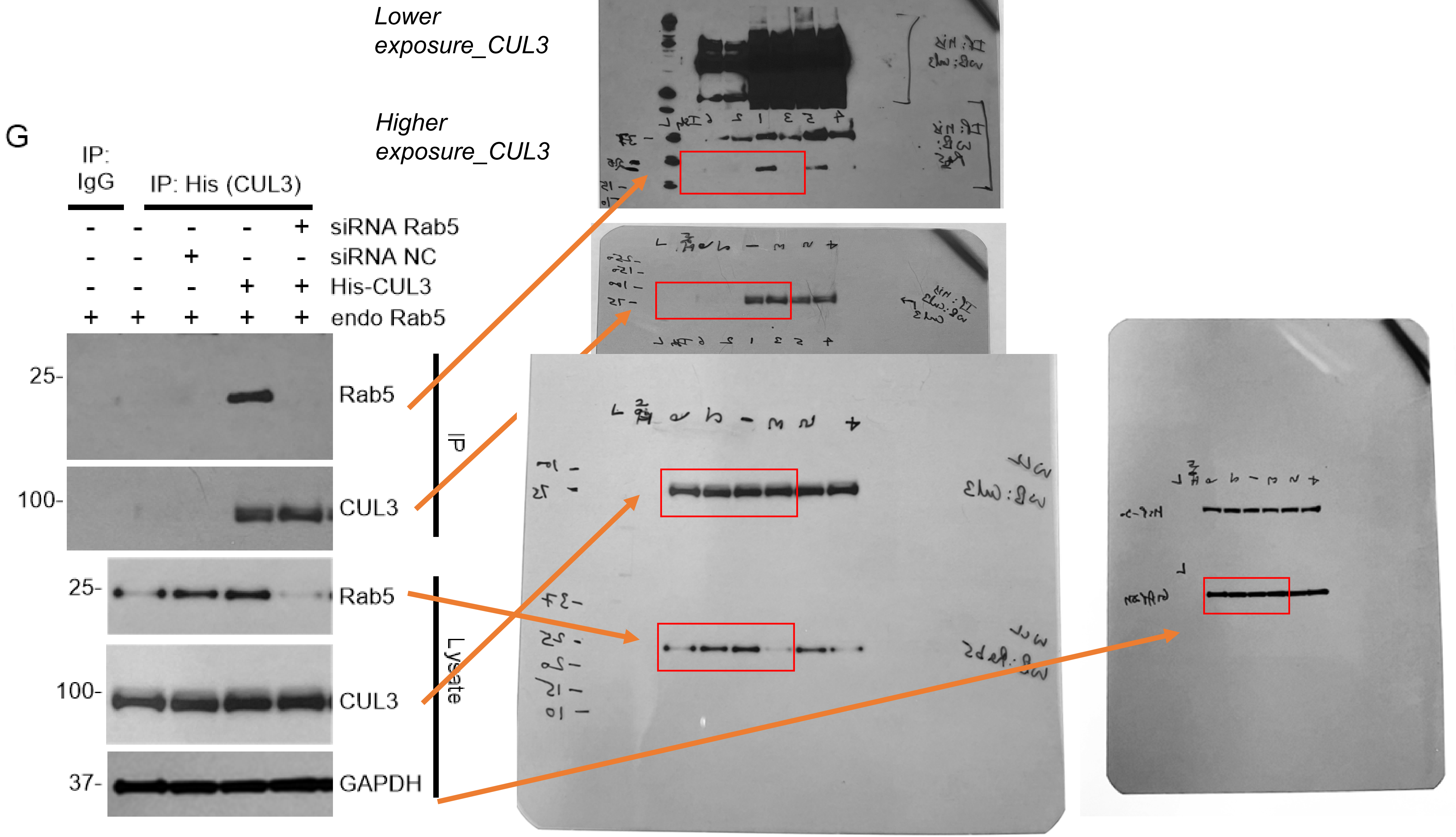

Figure 8C Full unedited blots for Figure 8C

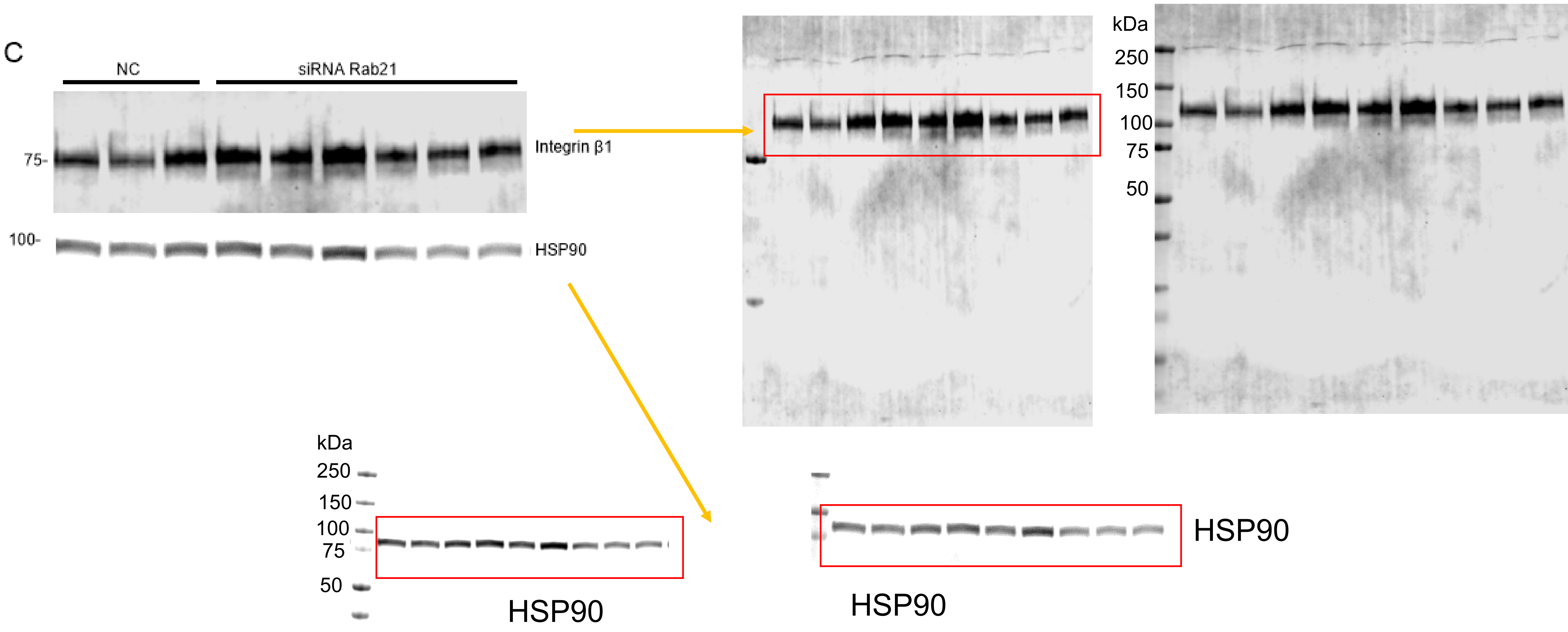

Same membranes,
Included channel 2 to reveal all ladder marks
